# The Impact of a Tablet App on Adherence to American Heart Association Guidelines During Simulated Pediatric Cardiopulmonary Resuscitation: Randomized Controlled Trial

**DOI:** 10.2196/17792

**Published:** 2020-05-27

**Authors:** Johan N Siebert, Laurence Lacroix, Aymeric Cantais, Sergio Manzano, Frederic Ehrler

**Affiliations:** 1 Department of Pediatric Emergency Medicine Geneva Children’s Hospital Geneva University Hospitals Geneva Switzerland; 2 Faculty of Medicine Geneva University Geneva Switzerland; 3 Pediatric Emergency Department University Hospital of Saint-Etienne Saint-Etienne France; 4 Diagnostic Department Geneva University Hospitals Geneva Switzerland

**Keywords:** biomedical technologies, mobile apps, emergency medicine, pediatrics, resuscitation, guideline adherence

## Abstract

**Background:**

Evidence-based best practices are the cornerstone to guide optimal cardiopulmonary arrest resuscitation care. Adherence to the American Heart Association (AHA) guidelines for cardiopulmonary resuscitation (CPR) optimizes the management of critically ill patients and increases their chances of survival after cardiac arrest. Despite advances in resuscitation science and survival improvement over the last decades, only approximately 38% of children survive to hospital discharge after in-hospital cardiac arrest and only 6%-20% after out-of-hospital cardiac arrest.

**Objective:**

We investigated whether a mobile app developed as a guide to support and drive CPR providers in real time through interactive pediatric advanced life support (PALS) algorithms would increase adherence to AHA guidelines and reduce the time to initiation of critical life-saving maneuvers compared to the use of PALS pocket reference cards.

**Methods:**

This study was a randomized controlled trial conducted during a simulation-based pediatric cardiac arrest scenario caused by pulseless ventricular tachycardia (pVT). A total of 26 pediatric residents were randomized into two groups. The primary outcome was the elapsed time in seconds in each allocation group from the onset of pVT to the first defibrillation attempt. Secondary outcomes were time elapsed to (1) initiation of chest compression, (2) subsequent defibrillation attempts, and (3) administration of drugs, including the time intervals between defibrillation attempts and drug doses, shock doses, and the number of shocks. All outcomes were assessed for deviation from AHA guidelines.

**Results:**

Mean time to the first defibrillation attempt (121.4 sec, 95% CI 105.3-137.5) was significantly reduced among residents using the app compared to those using PALS pocket cards (211.5 sec, 95% CI 162.5-260.6, *P*<.001). With the app, 11 out of 13 (85%) residents initiated chest compressions within 60 seconds from the onset of pVT and 12 out of 13 (92%) successfully defibrillated within 180 seconds. Time to all other defibrillation attempts was reduced with the app. Adherence to the 2018 AHA pVT algorithm improved by approximately 70% (*P*=.001) when using the app following all CPR sequences of action in a stepwise fashion until return of spontaneous circulation. The pVT rhythm was recognized correctly in 51 out of 52 (98%) opportunities among residents using the app compared to only 19 out of 52 (37%) among those using PALS cards (*P*<.001). Time to epinephrine injection was similar. Among a total of 78 opportunities, incorrect shock or drug doses occurred in 14% (11/78) of cases among those using the cards. These errors were reduced to 1% (1/78, *P*=.005) when using the app.

**Conclusions:**

Use of the mobile app was associated with a shorter time to first and subsequent defibrillation attempts, fewer medication and defibrillation dose errors, and improved adherence to AHA recommendations compared with the use of PALS pocket cards.

## Introduction

Pediatric cardiac arrest is a high-risk, low-frequency event associated with death or severe neurological sequelae in survivors. It requires immediate recognition and care by skilled health providers. Recent studies show that pediatric in-hospital cardiac arrest (p-IHCA) affects 7100-8300 children per year in the United States [1], of which 14% occur in pediatric emergency departments (PEDs) [2]. Pediatric out-of-hospital cardiac arrest (p-OHCA) accounts for a further 7037 children brought to US PEDs by emergency medical services each year [3]. Despite advances in resuscitation science and survival improvement over the last decades, survival remains low, with only approximately 38% of children surviving to hospital discharge after p-IHCA, and 6%-20% after p-OHCA [3,4]. Evidence-based best practices are the cornerstone for the guidance of optimal cardiopulmonary arrest resuscitation care. High-quality cardiopulmonary resuscitation (CPR), according to the American Heart Association (AHA) life-support guidelines, is associated with a successful return of spontaneous circulation (ROSC), improved survival after hospital discharge, and good neurological outcomes [5]. Deviation from recommended procedures is associated with a reduced likelihood of survival from cardiac arrest [6].

While adherence to AHA guidelines in emergency departments has been described for adults, there are limited data for PEDs [7]. Reference tools for pediatric emergency physicians to handle pediatric CPR according to AHA guidelines are available on reference pocket cards. Unfortunately, health care providers frequently do not perform resuscitation according to guidelines, despite cognitive aids [8] and AHA life-support training courses, such as basic life support (BLS) and pediatric advanced life support (PALS). Suboptimal quality of CPR is still commonly encountered for both adult and pediatric patients [9].

New resuscitation strategies using information technologies and devices aiming to improve both in- and out-of-hospital CPR have been assessed to ensure adherence to AHA guidelines [10-16]. Nevertheless, research in this area remains scarce, especially in pediatrics, and studies assessing the impact of information technology on p-IHCA management and improved pediatric CPR outcomes are necessary. In a previous randomized trial, we found that adherence to PALS algorithms when adapted on Google Glass was improved with a significant reduction of errors and deviations in defibrillation doses by 53% when compared to the use of pocket reference cards [17]. However, time to the first defibrillation attempt and adherence to AHA guidelines to other critical resuscitation endpoints in terms of time and drug-dose delivery were not improved using the glasses. The complexity of interacting while wearing glasses, as well as the limits of the system to situate the current action in the whole resuscitation process and their small size, were major limitations to their potential use in p-IHCA. Thus, we have developed a new mobile app—the Guiding Pad app—from the ground up and dedicated it to tablets. It is intended as a guide to support and drive CPR providers in real-time conditions through interactive PALS algorithms enhanced with patient-centered cognitive aids.

Our objective was to investigate, in a simulated model, whether this app would increase adherence to AHA guidelines by reducing deviation and time to initiation of critical life-saving maneuvers during pediatric CPR compared to the use of PALS pocket reference cards.

## Methods

### Study Design

We conducted a prospective, randomized controlled trial in a tertiary PED (>33,000 consultations/year) with two parallel groups of voluntary pediatric residents. We compared time to the first defibrillation attempt and other critical resuscitation endpoints using a tablet app (Guiding Pad, group A) or AHA PALS conventional pocket reference cards (group B) during a standardized simulation-based pediatric cardiac arrest scenario. No changes were made to the app during the study.

The trial received a declaration of *no objection* by Swissethics and the Geneva Cantonal Ethics Committee, as the purpose of the study was to examine the effect of the intervention on health care providers. For the same reason, and according to the International Committee of Medical Journal Editors, a trial registration number was not required. The trial was conducted according to the principles of the Declaration of Helsinki and Good Clinical Practice guidelines, and in accordance with the CONSORT-EHEALTH (Consolidated Standards of Reporting Trials of Electronic and Mobile Health Applications and Online TeleHealth) guidelines (see Multimedia Appendix 1) [18] and the Reporting Guidelines for Health Care Simulation Research [19].

### Participants

Any physician performing a residency in pediatrics was eligible. Shift-working residents were randomly recruited on the day of the study using an alphabetical list to avoid preparation bias. Included participants benefited from a standardized 5-minute introduction course on the use of the tablet app. As BLS training is a requirement for residents at our institution, all participants had previously completed this course prior to study entry. Participation in a simulation in the past month was an exclusion criterion to avoid a recent training effect. Study participants were not involved in the study design, choice of outcome measures, or the execution of the study. No participant was asked for advice on the interpretation or writing of the study results. Participants were informed of the results after completion of the study.

### Randomization and Blinding

Residents were randomized using a single constant 1:1 allocation ratio determined with the web-based randomization software Sealed Envelope [20]. Written informed consent was obtained from each participant after full information disclosure prior to study participation. Blinding to the purpose of the study was maintained during recruitment to minimize preparation bias. Allocation concealment was managed with the software and was not released until the participant started the scenario.

### The Guiding Pad App

Unlike adults, cardiac arrest in children without prior cardiac disease is mainly due to asystole (40%) and pulseless electrical activity (24%) [2]. As ventricular fibrillation and pulseless ventricular tachycardia (pVT), namely shockable rhythms, have been identified in 27% of p-IHCA cases [21], we decided to use the pVT algorithm, as we considered that it would offer a greater opportunity to assess the multiple-step resuscitative skills set out in the AHA guidelines. The app was developed at Geneva University Hospitals using Angular, version 8, a development framework created by Google to build mobile and web apps. The AHA PALS algorithms were adapted for tablets following a user-centered and ergonomic-driven approach by computer scientists, senior pediatric emergency physicians, and ergonomists. The numerous steps of AHA PALS algorithms were split into stages. Each stage transposed to the tablet paralleled the informational content of a resuscitation step from the original algorithm. The algorithms thus obtained were set up in a manner similar to the PALS pocket references regarding the progression and sequence of actions along the original algorithms’ sequences. For instance, the complete pVT algorithm was designed to be as concise as possible without hindering proper progression along the algorithm. After completing a quick pediatric assessment triangle as the first step to recognize cardiac arrest and initiate CPR, including information on the weight or age of the patient, the app displays an initial statement about whether the pulse is present or not and the subsequent cardiac arrhythmias. Once selected, the screen then splits into two main sections.

The first section on the left-hand side of the screen displays an entire algorithm overview, thus allowing users to situate the stepwise resuscitation progress in real time along that algorithm. The current step of the resuscitation process is surrounded by a blinking red line that allows an immediate understanding of the current position within the algorithm. Each action already performed turns grey. However, the app provides the possibility for users to navigate back and forth through the algorithm at any time in order to select one of the resuscitation steps if needed.

**Figure 1:**
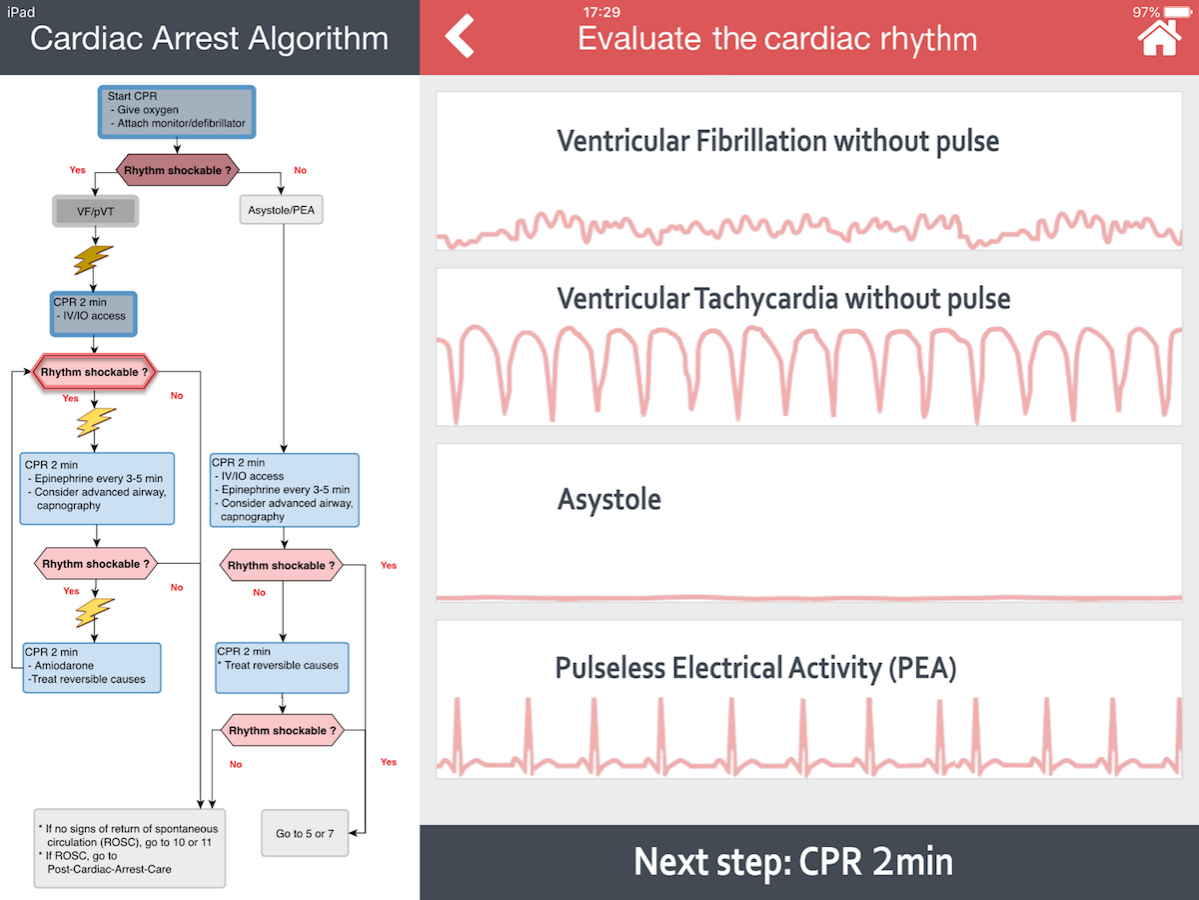
A screenshot of the Guiding Pad app. The left-hand side of the screen displays the American Heart Association (AHA) pediatric advanced life support (PALS) ventricular fibrillation (VF) and pulseless ventricular tachycardia (pVT) cardiac arrest algorithm. The current step (eg, determining the shockable status of the arrhythmia) of the resuscitation process is surrounded by a blinking red line. Past actions already accomplished are shown as shaded. At the top right-hand side of the screen, a color-coded title depicts the current step in progress. Below, four pulseless dysrhythmias are displayed; the provider selects the right one under consideration. At the bottom right-hand side, a footer helps to anticipate the next cardiopulmonary resuscitation (CPR) step. IO: intraosseous; IV: intravenous.

**Figure 2:**
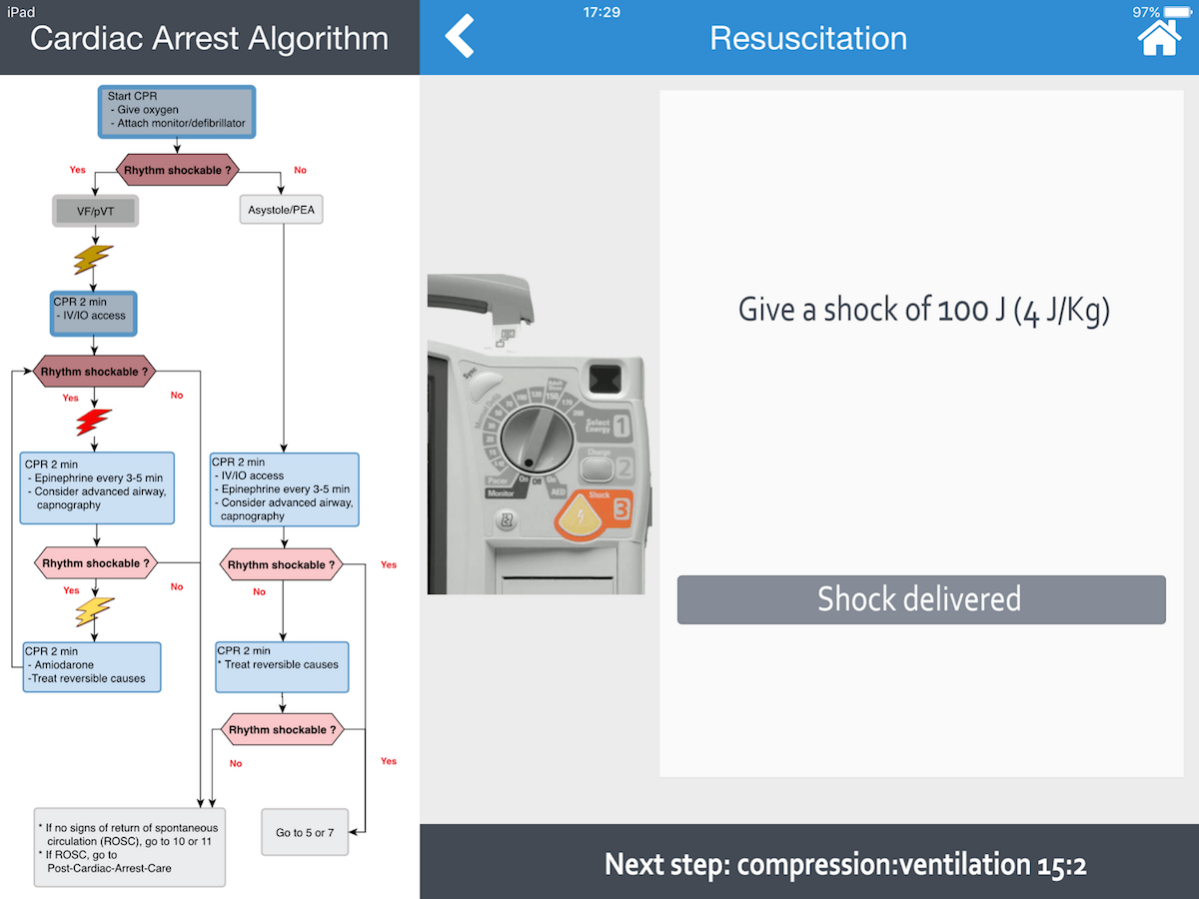
Screenshot of the Guiding Pad app. The left-hand side of the screen displays the American Heart Association (AHA) pediatric advanced life support (PALS) ventricular fibrillation (VF) and pulseless ventricular tachycardia (pVT) cardiac arrest algorithm. The current step (eg, defibrillation) of the resuscitation process is displayed with a red lightning bolt. Past actions already accomplished are shown as shaded. On the right-hand side of the screen, the weight-based shock dose to deliver is displayed with a picture of a manual defibrillator (Philips HeartStart MRx Biphasic Defibrillator). Once delivered, clicking the “Shock delivered” button validates the action and allows the user to proceed to the next step. At the bottom right-hand side, a footer helps to anticipate the next compression:ventilation step. CPR: cardiopulmonary resuscitation; IO: intraosseous; IV: intravenous; PEA: pulseless electrical activity.

The second section on the right-hand side of the screen displays the following elements:

1. A color-coded title allowing direct identification of each step in progress.
2. Cognitive aids helping with decision making, such as a distinctive illustration of cardiac rhythms (see Figure 1) or the shock dose to deliver with a picture of a manual defibrillator (Philips HeartStart MRx Biphasic Defibrillator, Philips Medical Systems) (see Figure 2).
3. A detailed and clickable list of actions to perform in a stepwise manner.
4. A footer to preview the next step.

**Figure 3:**
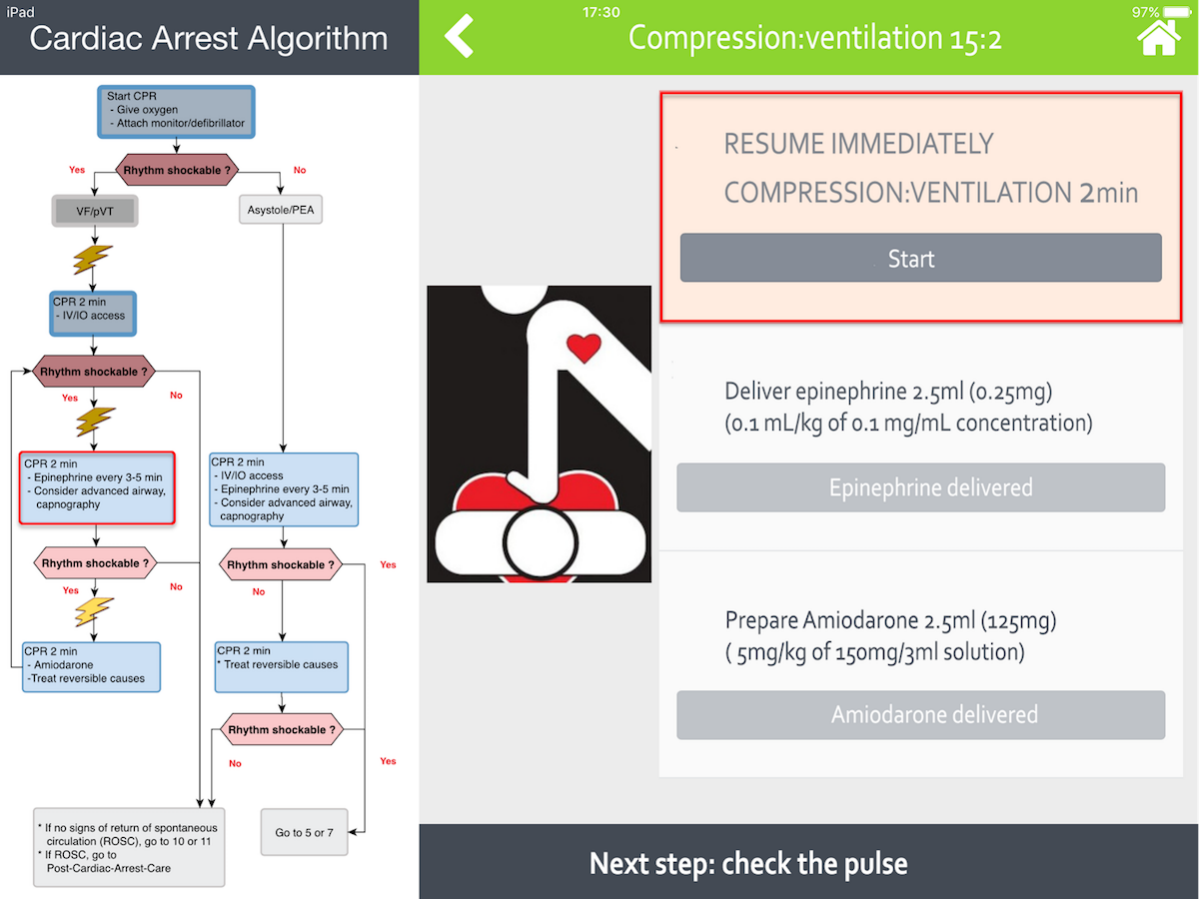
Screenshot of the Guiding Pad app. The left-hand side of the screen displays the American Heart Association (AHA) pediatric advanced life support (PALS) ventricular fibrillation (VF) and pulseless ventricular tachycardia (pVT) cardiac arrest algorithm. On the right-hand side of the screen, the sequence of actions to be taken are displayed in a stepwise manner to facilitate accurate progression along the algorithm. The current action (eg, to resume compression and ventilation) is brought to the attention of the provider by a red-box warning and requires validation by a simple click. Once completed, the next action will be to deliver the weight-based epinephrine dose automatically calculated by the app and then to prepare amiodarone. The next step shown at the bottom right-hand side will be to check the pulse. CPR: cardiopulmonary resuscitation; IO: intraosseous; IV: intravenous; PEA: pulseless electrical activity.

Each action prompts the provider either to perform a choice (ie, choose the correct arrhythmia among several propositions) or to validate an action to be executed (eg, a drug-dose administration), which is brought to the attention of the provider by a red-box warning (see Figure 3). Weight-based drug doses are automatically calculated by an in-built engine already used in another evidence-based app that was assessed in a multicenter randomized controlled trial for in-hospital emergency drug delivery [22]. Shock doses using Philips HeartStart MRx Biphasic Defibrillator are automatically calculated based on patients’ weights or ages. Each cycle of chest compression-ventilation is timed by a countdown clock displayed on the screen. In the case of rhythm change, the user can easily navigate across the multiple PALS algorithms (bradycardia, supraventricular tachycardia with poor perfusion, etc) at any time. All actions performed by the provider are automatically saved in log files to preserve information that can be retrieved at any time for debriefing or medicolegal purposes.

### Procedures

**Figure 4:**
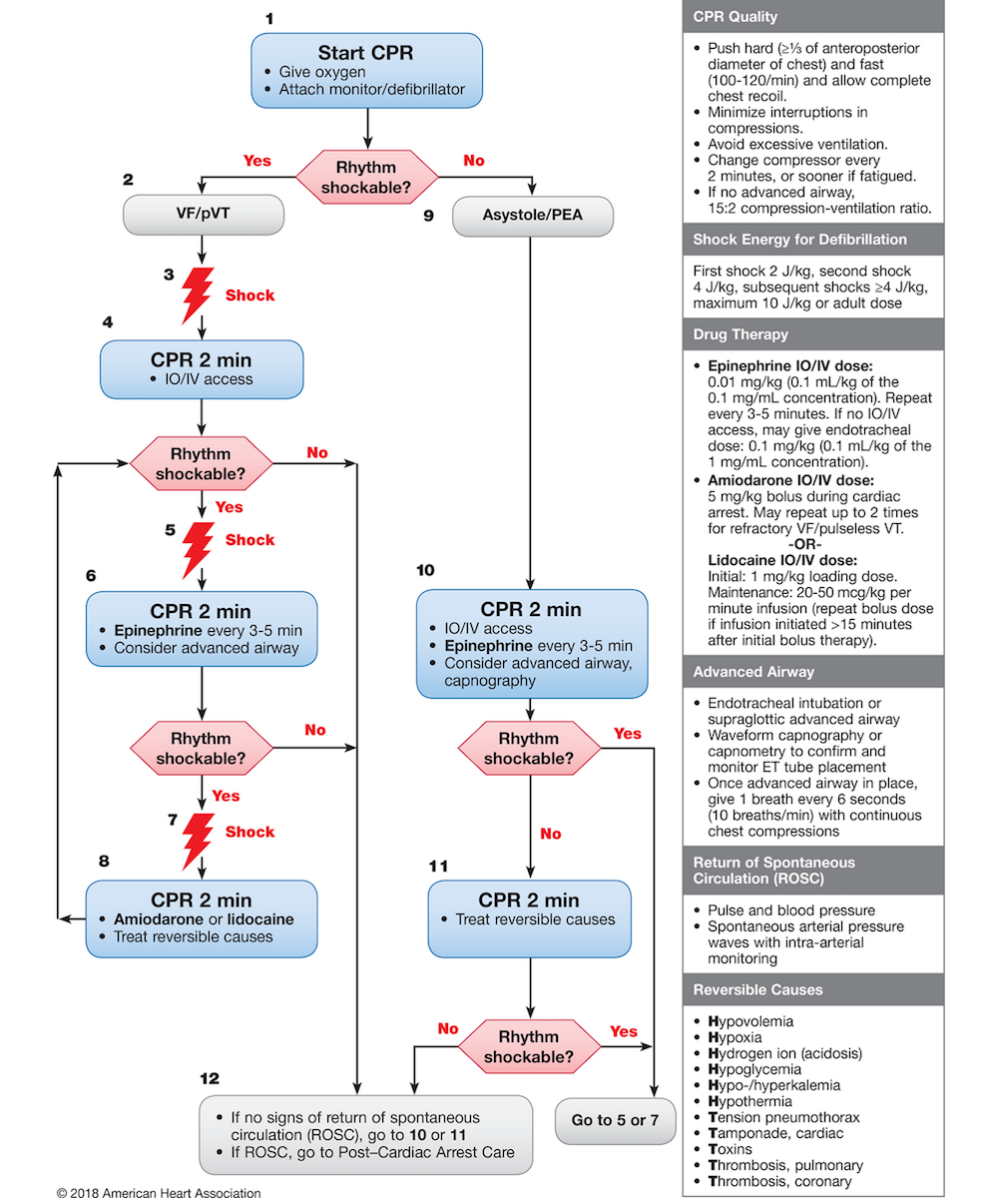
American Heart Association (AHA) pediatric cardiac arrest algorithm: 2018 update (Duff et al, 2018). CPR: cardiopulmonary resuscitation; ET: endotracheal tube; IO: intraosseous; IV: intravenous; PEA: pulseless electrical activity; pVT: pulseless ventricular tachycardia; VF: ventricular fibrillation.

On the day of participation, each resident completed an anonymous survey on basic demographic information, professional length of clinical experience, and PALS training. After random allocation, each participant received a standardized 5-minute training session on how to use the app. Participants were then asked to perform a 15-minute, highly realistic, scripted CPR scenario on a high-fidelity manikin (SimJunior; Laerdal Medical). The scenario was standardized to strictly follow the 2018 AHA pediatric pVT algorithm (see Figure 4) and was performed on the same high-fidelity manikin already primed with vital signs appropriate for the scenario (see Multimedia Appendix 2). It was conducted in situ in the PED shock room to increase realism, thus allowing participants to make use of real resources in the actual environment where they were expected to handle cardiac arrest. All participants in group B were offered the possibility to hold PALS pocket reference cards in their hands throughout the entire scenario. Whether they referred to them or not was left to their discretion, similar to real-life settings. No interactions occurred between participants and investigators. The simulation involved the participating resident and a resuscitation team comprising three study team members (ie, a PED registered nurse and two medical students) to assist with resuscitation through drug preparation, chest compression, and bag-valve-mask ventilation. Study team members had no role in decision making to achieve ROSC. A PALS instructor (ie, a pediatric emergency physician) who was not a member of the resuscitation team operated the simulator. To be consistent with the 2018 AHA pediatric cardiac arrest algorithm [23] and to standardize the scenario, defibrillation doses of 2 Joules per kg for the first attempt, and 4 Joules per kg for the subsequent second, third, and fourth attempts, were expected (see Figure 4).

**Figure 5:**
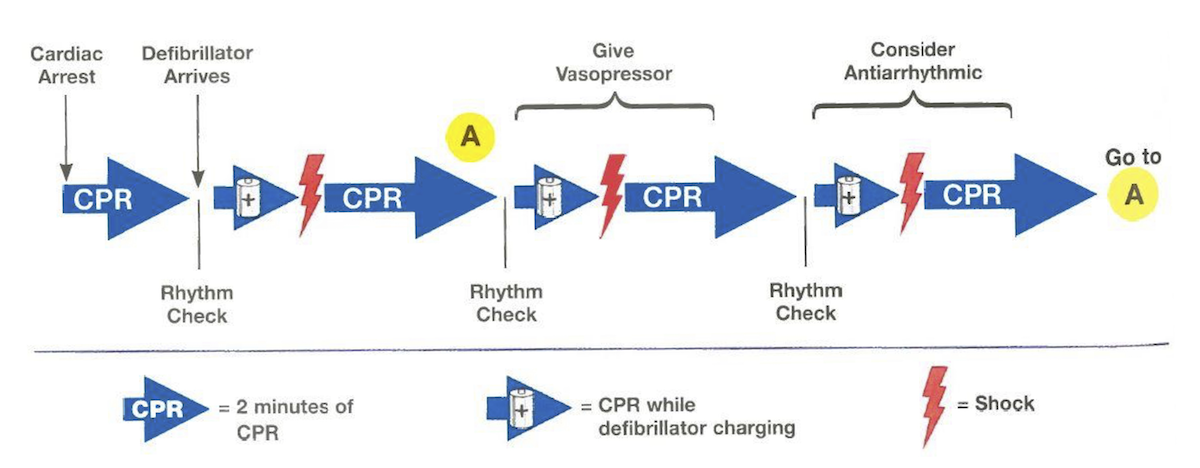
Summary of the ventricular fibrillation and pulseless ventricular tachycardia (pVT) cardiac arrest sequence. This original illustration is from the eBook edition of the Pediatric Advanced Life Support (PALS) Instructor Manual, published by the American Heart Association (AHA), 2015. CPR: cardiopulmonary resuscitation.

Epinephrine and amiodarone drug doses had to be given just before or just after the second or third shock attempts, respectively (see Figure 5). The ROSC as demonstrated by a palpable pulse and signs of regaining consciousness corresponded to the end of the scenario.

### Outcomes

The primary outcome was the delay (in seconds) in each allocation group from the end of the clinical statement given by the study investigator to the first defibrillation attempt, as the expected survival advantage from early CPR can be significantly affected by a subsequent delay in defibrillation [24,25]. Secondary outcomes were the delay (in seconds) to initiation of chest compression; time to subsequent defibrillation attempts; time to administration of epinephrine and amiodarone; time interval (in seconds) between defibrillation attempts, drug doses, shock doses, and number of shocks; and perceived stress and satisfaction scores after completion of the scenario, as measured by a questionnaire using 10-point Likert scales (see Multimedia Appendix 3). The AHA recommends five cycles of chest compression (approximately 2 minutes) between each defibrillation attempt. The time spent by participants to perform chest compressions by compression cycles was defined as the hands-on time and was measured in seconds with a chronometer. All these outcomes were assessed for deviation from AHA guidelines.

### Methods of Measurement and Data Collection

All actions (ie, primary and secondary outcomes) performed by the resident during the scenario were independently recorded by two trained investigators blinded to each other’s records during the simulation, thus allowing an accurate assessment of timing and sequencing of actions and avoiding assessment bias. In the case of disagreement, a third independent evaluator helped reach a consensus. Data were manually retrieved and entered into a Microsoft Excel spreadsheet, version 16 (Microsoft Corporation‬). Unaccomplished actions were left blank and time was not assigned. Residents’ privacy was preserved. Only the study investigators had access to the data.

### Statistical Analysis

Power calculations were based on the detection of a 30-second decrease in time to the first defibrillation attempt between the two independent groups. A previous study has shown a mean time to first defibrillation of 92 seconds with an SD of 23 seconds [26]. Assuming a similar SD in each group of our study, 10 participants per group had to be recruited to provide the trial with 80% power at a two-sided alpha level of .05. To prevent a potential loss of power due to misspecification of assumptions, 13 participants were recruited per group, giving a total sample size of 26 participants.

For the primary analysis, we first evaluated the time elapsed between the onset of pVT and first defibrillation attempt. The Shapiro-Wilks test was used for normality analysis of the parameters. As most of the continuous variables were normally distributed, means and SDs with their 95% CIs were reported. Nonnormally distributed variables were analyzed using a Mann-Whitney test. Frequencies were reported as percentages. We used *t* tests to compare independent groups. No paired data were compared. Kaplan-Meier curves for time elapsed between the onset of pVT and first defibrillation attempt were estimated and compared using the log-rank (Mantel-Cox) test for bivariate survival analysis.

For the secondary analysis, we evaluated the time elapsed between the onset of pVT to subsequent defibrillation attempts and the delivery of both drugs. For normally distributed variables, means and SDs with 95% CI were reported. Nonnormally distributed variables were analyzed using a Mann-Whitney test. Frequencies were reported as percentages. We used *t* tests to compare independent groups. No paired data were compared. Kaplan-Meier curves for time elapsed between the onset of pVT and subsequent defibrillation attempts and delivery of both drugs were also estimated and compared using the log-rank (Mantel-Cox) test for bivariate survival analysis. Errors in cycles of chest compression-ventilation were measured as the deviation in percent from the experimental time spent in seconds compared to the 2-minute duration recommended by the AHA. Incorrect defibrillation or drug doses were measured as a deviation from the amount of energy delivered in Joules or drug doses in milliliters compared to AHA recommendations. A chi-square test was used to assess the relationship between absolute errors in defibrillation and drug doses expressed as categorical variables. Incorrect defibrillation mode was also measured. Absolute deviations were also analyzed. The mean (SD) difference in deviation obtained with each method was reported with a 95% CI. A *t* test for unpaired data was used to compare interventions. Mean differences were reported by randomized group. Univariate linear regression analyses with 95% CI were performed to assess whether time to initiation of chest compression, defibrillation attempts, and drug delivery were associated with the number of postgraduate years or prior resuscitation experience as a provider in real-life and simulated environments. Means and SDs were determined for the perceived stress and satisfaction scores of individuals derived from the Likert-scale questionnaire and reported with descriptive statistics. A *P* value less than .05 was considered significant.

Interrater reliability was assessed by two observers who independently evaluated each resident’s performance. Interrater reliability scores were calculated using the Cohen kappa coefficient for the shock and drug-dose errors. As the remaining outcomes were continuous variables, the Bland-Altman method was used to plot the difference of values reported by both observers against the mean value for each outcome. The limits of agreement were assessed by the interval of SD 1.96 of the measurement differences on either side of the mean difference. The null hypothesis that there was no difference, on average, between both reviewers was tested using a *t* test. The mean difference was reported with its 95% CI. Additionally, the intraclass correlation coefficients for times to each critical endpoint were assessed, assuming that raters were comprised of a sample from a larger population of possible raters. GraphPad Prism, version 8 (GraphPad Software), and SPSS, version 15.0 (SPSS Inc), were used for graph figures and to perform descriptive and statistical analyses.

## Results

### Study Participants

**Figure 6:**
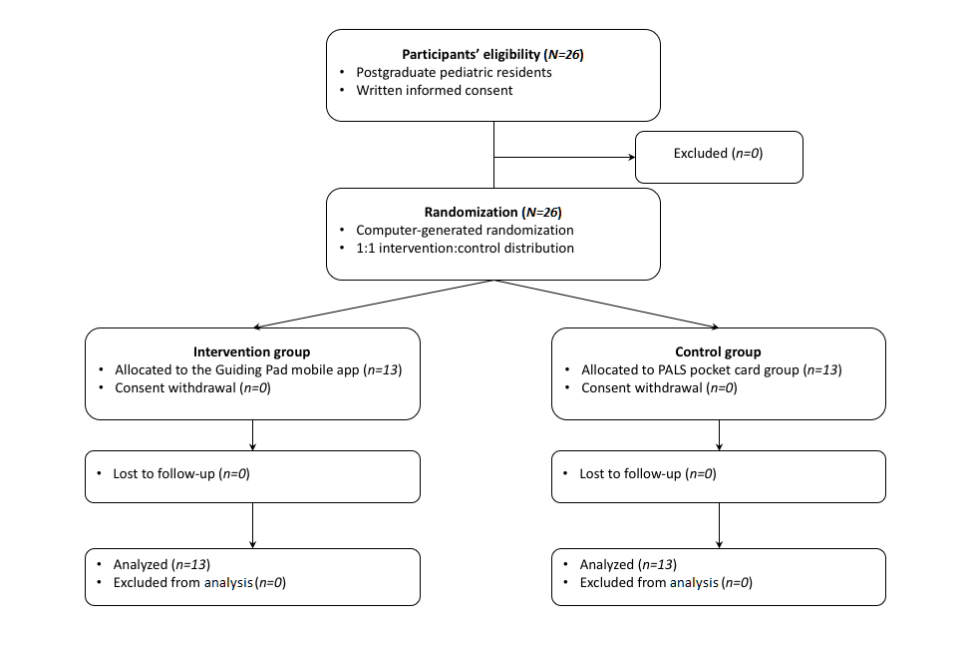
Trial flowchart. PALS: pediatric advanced life support.

From August 30 to October 17, 2019, 26 pediatric residents were assessed for eligibility and randomly assigned to either the Guiding Pad app group (group A) (n=13) or the PALS pocket card group (group B) (n=13), without any dropouts or missing data (see Figure 6). Baseline characteristics were balanced in the two groups (see Table 1). In particular, we observed no statistically significant difference between the ages of both randomization arms (ie, no bias in randomization). We observed perfect interrater agreement for the scoring of the pVT scenario (see Table S1 in Multimedia Appendix 4, Table S2 in Multimedia Appendix 5, and Figure S1 in Multimedia Appendix 6).

**Table 1:** Participants’ demographics and clinical characteristics.

Demographics and clinical characteristics	Randomization arm	
	Guiding Pad (n=13)	PALS^a^ pocket cards (n=13)
Age in years^b^, mean (SD)	31.2 (5.5)	29.0 (2.2)
Sex (female), n (%)	9 (69)	10 (77)
Years of residency, mean (SD)	3.7 (2.3)	3.5 (0.9)
Number of basic life support providers among residents, n (%)	13 (100)	13 (100)
Number of PALS providers among residents, n (%)	9 (69)	11 (85)
Level of self-confidence^c^ in following American Heart Association guidelines, mean (SD)	2.9 (1.1)	3.5 (0.7)
Number of residents having been enrolled in more than five resuscitations in the past, n (%)	4 (31)	7 (54)
Prior simulation-based resuscitations, mean (SD); total	6.5 (6.2); 84	4.8 (3.5); 62
Prior real-world cardiopulmonary resuscitations, mean (SD); total	9.2 (21.4); 120	7.8 (7.3); 102
Prior use of a manual defibrillator in either real-world or simulated environments, n (%)	10 (77)	9 (69)
Months since last manual-mode defibrillator use in either real-world or simulated environments, mean (SD)	9.5 (10.2)	13.9 (17.8)

^a^PALS: pediatric advanced life support.

^b^The age difference between the randomization arms was not statistically significant.

^c^Self-confidence was measured on a scale of 1 (not confident) to 5 (very confident).

### Time to Critical Resuscitation Endpoint

**Figure 7:**
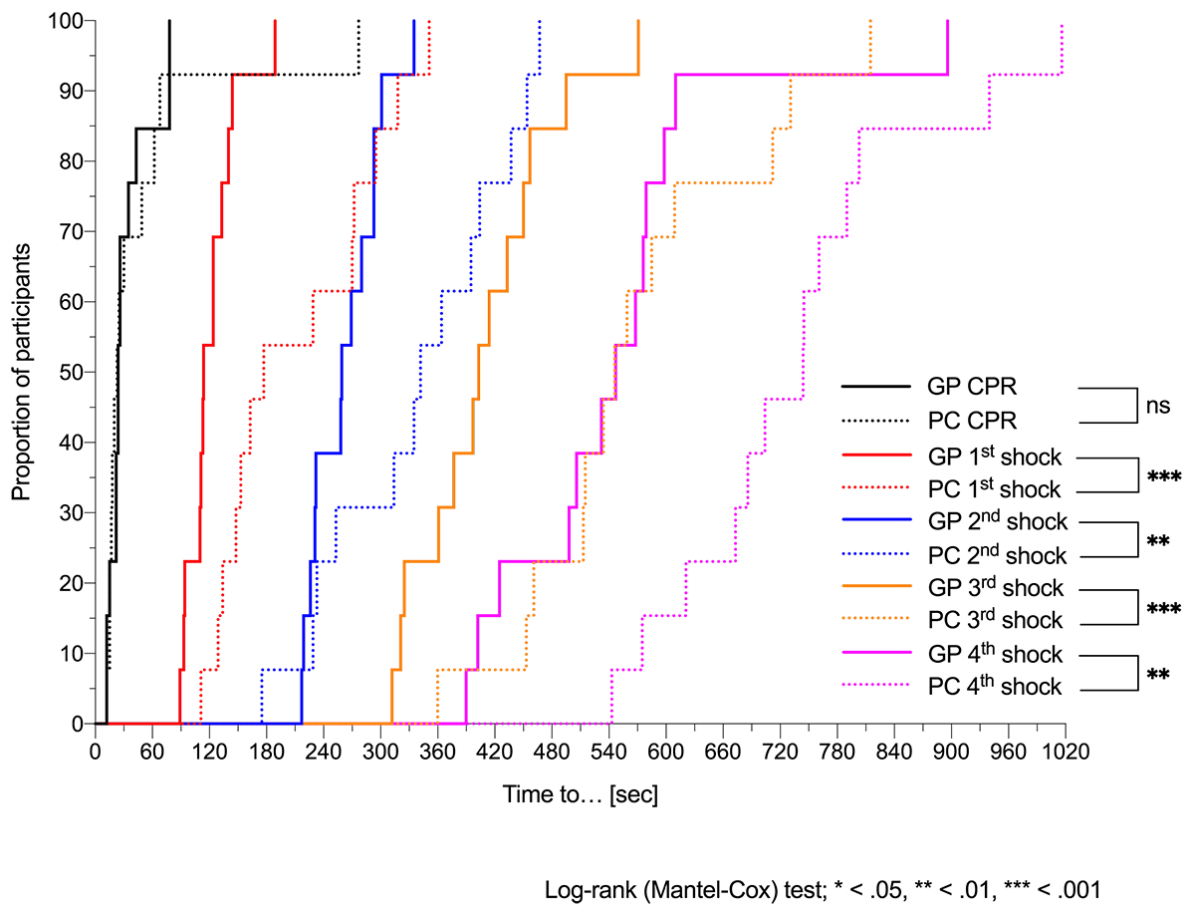
Time to cardiopulmonary resuscitation (CPR) and defibrillation attempts. Kaplan-Meier curves of time elapsed between the onset of simulated pulseless ventricular tachycardia (pVT) and initiation of chest compression (ie, CPR) for the first, second, third, and fourth defibrillation attempts for residents using the Guiding Pad (GP) app vs conventional pediatric advanced life support (PALS) pocket cards (PCs). Log-rank (Mantel-Cox) test comparing curves: χ^2^=0.0 and *P*=.97 for CPR; χ^2^=13.9 and *P*<.001 for the first defibrillation attempt; χ^2^=8.9 and *P*=.003 for the second defibrillation attempt; χ^2^=13.3 and *P*<.001 for the third defibrillation attempt; and χ^2^=9.5 and *P*=.002 for the fourth defibrillation attempt. ns: not significant.

Using the Guiding Pad app, 11 residents out of 13 (85%) initiated chest compressions within 60 seconds of the onset of pVT (9/13, 69%, within 30 sec), and 12 out of 13 (92%) successfully defibrillated within 180 seconds (see Figure 7). Mean time elapsed between the onset of pVT and first defibrillation attempt was 121.4 seconds (SD 26.7).

**Figure 8:**
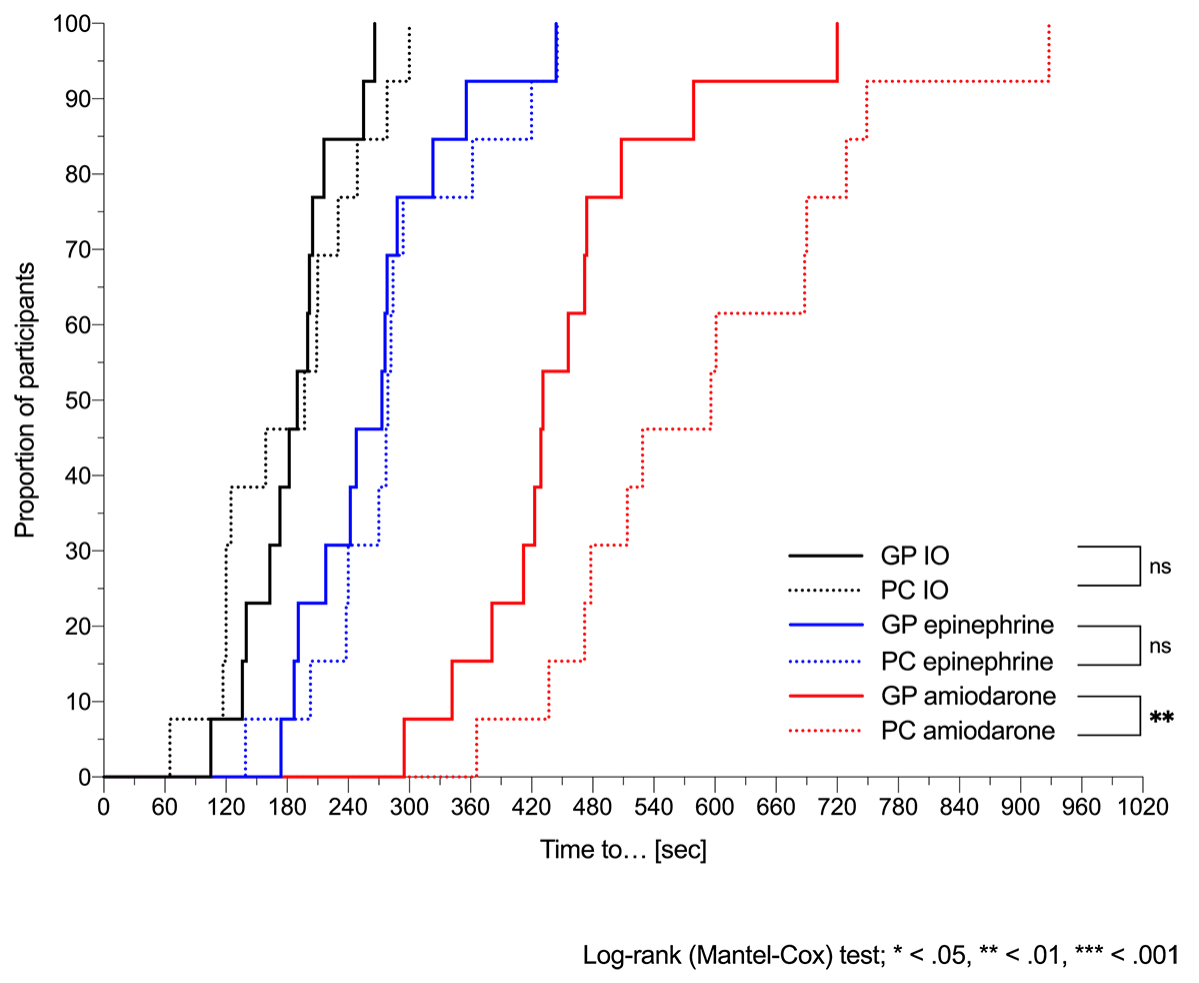
Time to intraosseous (IO) route and drug delivery. Kaplan-Meier curves of time elapsed between the onset of simulated pulseless ventricular tachycardia (pVT) and the IO insertion, epinephrine, and amiodarone delivery for residents using the Guiding Pad (GP) app vs conventional pediatric advanced life support (PALS) pocket cards (PCs). Log-rank (Mantel-Cox) test comparing curves: χ^2^=0.4 and *P*=.55 for the IO route; χ^2^=0.6 and *P*=.44 for epinephrine; and χ^2^=7.5 and *P*=.006 for amiodarone. ns: not significant.

With PALS pocket cards, out of 13 residents, 10 (77%) started compressions within 60 seconds, 1 (8%) started compressions 277 seconds after onset of pVT, and 6 (46%) failed to discharge the defibrillator within 180 seconds. Mean time from initiation of chest compression to the first shock was significantly reduced for residents using the app (89.3 sec) than for those using the PALS pocket cards (163 sec; *P*=.002). Mean times to other critical resuscitation endpoints are summarized in Table 2. All defibrillation attempts, as well as amiodarone administration, were delivered significantly earlier in group A than in group B. However, the app was unable to speed up the delay before intraosseous access and epinephrine delivery (see Table 2 and Figure 8). We sought to analyze, in both groups, the difference in mean time to first defibrillation attempts between residents with or without previous defibrillation experience in either real-world or simulated environments, but with our small sample size we did not find any difference (with the app: 124.1 vs 112.3 sec, *P*=.53; without the app: 268.8 vs 186.1 sec, *P*=.09).

**Figure 9:**
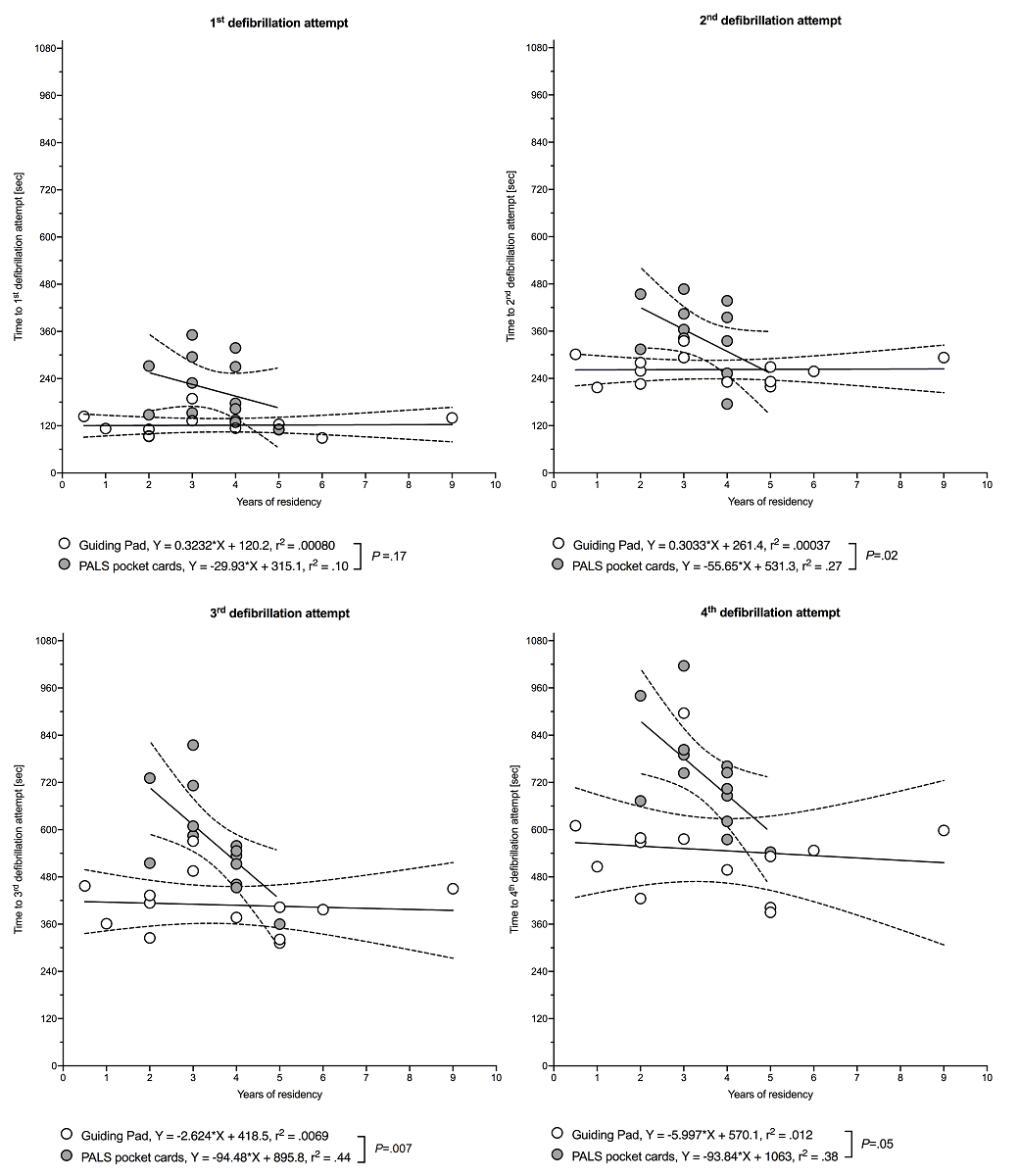
Association between time to defibrillation attempts and years of residency. Data are shown as a regression line (solid) with 95% CI (dashed lines). *P* values and r^2^ values are based on simple linear regression analysis. White (Guiding Pad app) and grey (pediatric advanced life support [PALS] pocket cards) open circles denote each individual value.

At the time of the study, 24 participants out of 26 (92%) were residents with more than one year of pediatric training (ie, postgraduate years). In a simple linear regression model, using the app was associated with a significant or borderline significant reduction in time to defibrillation attempts, regardless of the postgraduate years, and less scattered delays around the mean defibrillation time than when using the pocket cards (see Figure 9).

**Figure 10:**
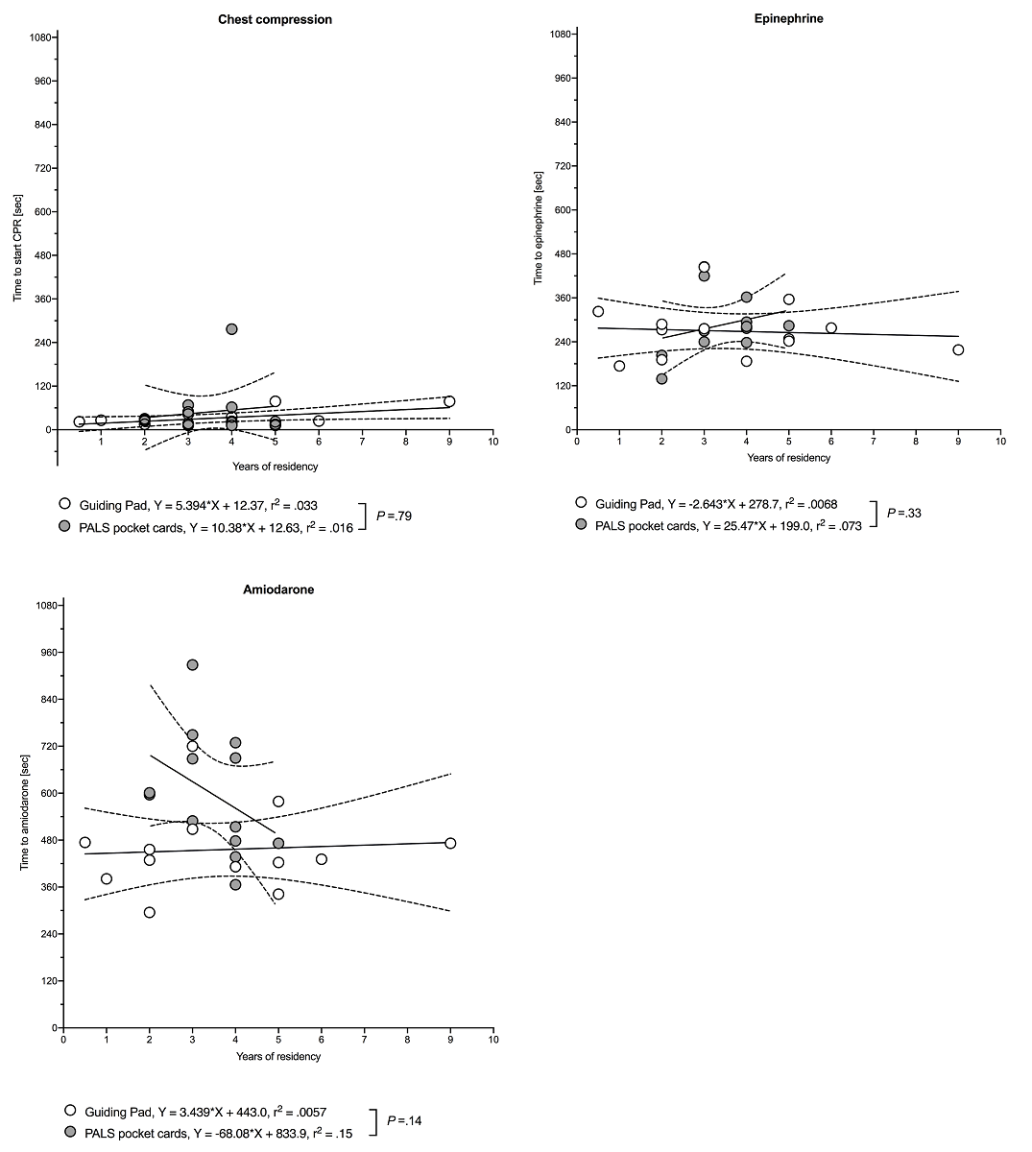
Association between time to chest compression or drug delivery and years of residency. Data are shown as a regression line (solid) with 95% CI (dashed lines). *P* values and r^2^ values are based on simple linear regression analysis. White (Guiding Pad app) and grey (pediatric advanced life support [PALS] pocket cards) open circles denote each individual value. CPR: cardiopulmonary resuscitation.

In group B, time to defibrillation attempts was inversely associated with the number of postgraduate years. In both groups, we observed no correlation between the time to initiation of chest compression or time to drug delivery and postgraduate years (see Figure 10). Moreover, we observed no relationship between previous CPR experience expressed as the number of prior CPR attempts on either a patient or a manikin and times to initiation of CPR, defibrillation attempts, or drug delivery (see Figure S2 in Multimedia Appendix 7 and Figure S3 in Multimedia Appendix 8).

**Table 2:** Mean time to critical resuscitation endpoints.

Outcome	Mean time^a^ for Guiding Pad app group (n=13), seconds		Mean time^a^ for PALS^b^ pocket cards group (n=13), seconds		Time difference^c^, seconds	*P* value
	Mean (SD)	95% CI	Mean (SD)	95% CI		
Start chest compression	32.1 (22.1)	18.7-45.4	48.5 (71.1)	5.6-91.5	16.5	.91^d^
First defibrillation attempt	121.4 (26.7)	105.3-137.5	211.5 (81.2)	162.5-260.6	90.1	<.001
Intraosseous route	187.2 (45.4)	159.7-214.6	183.0 (71.3)	139.9-226.1	4.2	.86
Second defibrillation attempt	262.5 (36.7)	240.3-284.7	338.6 (93.8)	282.0-395.3	76.1	.01
Epinephrine	269.1 (74.8)	223.8-314.3	287.2 (82.9)	237.1-337.2	18.1	.56
Third defibrillation attempt	408.9 (74.1)	364.1-453.7	568.7 (124.4)	493.5-643.9	159.8	<.001
Amiodarone	455.5 (106.9)	391.0-520.1	598.2 (154.7)	504.7-691.7	142.7	.01
Fourth defibrillation attempt	548.2 (127.6)	471.1-625.4	738.5 (132.9)	658.2-818.9	190.3	<.001^d^

^a^The mean time in each allocation group refers to the delay in seconds from the end of the clinical statement given by the study investigator to each critical resuscitation endpoint.

^b^PALS: pediatric advanced life support.

^c^Time difference represents the absolute time difference between mean PALS pocket cards group and Guiding Pad app group outcomes.

^d^Mann-Whitney test.

### Errors and Deviations From the AHA pVT Algorithm

Errors and deviations from the AHA pVT algorithm are summarized in Table 3 and in Table S1 in Multimedia Appendix 4. The entire pVT algorithm was followed correctly in a stepwise fashion until ROSC by 12 out of 13 (92%) residents in group A and only 3 out of 13 (23%) residents in group B (*P*=.001) (see Table 4). Importantly, the pVT rhythm was recognized correctly in 51 out of 52 opportunities (98%) by residents using the app, but in only 19 out of 52 opportunities (37%) of those using the pocket cards (*P*<.001).

**Table 3:** Errors and deviations from the American Heart Association (AHA) pulseless ventricular tachycardia (pVT) algorithm with respect to critical resuscitation endpoints.

Critical resuscitation endpoint	AHA recommended dose	Guiding Pad app (n=13)			PALS^a^ pocket cards (n=13)		
		Dose, mean (SD)	95% CI	Dose range	Dose, mean (SD)	95% CI	Dose range
First defibrillation attempt (J/kg)	2.00	2.00 (0)	2.00-2.00	2.00-2.00	1.97 (0.76)	1.51-2.43	0.60-4.00
Second defibrillation attempt (J/kg)	4.00	3.85 (0.55)	3.51-4.18	2.00-4.00	3.62 (0.96)	3.04-4.20	1.00-4.00
Epinephrine 0.1 mg/mL (mL/kg)	0.10	0.10 (0)	0.10-0.10	0.10-0.10	0.09 (0.03)	0.08-0.11	0.001-0.10
Third defibrillation attempt (J/kg)	4.00	4.15 (0.55)	3.82-4.49	4.00-6.00	3.87 (1.57)	2.92-4.82	0.52-6.00
Amiodarone (mL/kg)	0.10	0.10 (0)	0.10-0.10	0.10-0.10	0.10 (0.01)	0.10-0.10	0.10-0.14
Fourth defibrillation attempt (J/kg)	4.00	4.31 (1.11)	3.64-4.98	4.00-8.00	5.45 (1.67)	4.44-6.46	4.00-8.00

^a^PALS: pediatric advanced life support.

**Table 4:** Errors and deviations from the American Heart Association (AHA) pulseless ventricular tachycardia (pVT) algorithm with respect to defibrillation and drug factors.

Defibrillation and drug factors	Guiding Pad app (n=13), n (%)	PALS^a^ pocket cards (n=13), n (%)
Correct number of shocks (N=52)	51 (98)	51 (98)
Error in shock or drug doses (N=78)	1 (1)	11 (14)^b^
pVT rhythm recognition (N=52)	51 (98)	19 (37)^c^
Correct AHA sequence (n=13)	12 (92)	3 (23)^d^

^a^PALS: pediatric advanced life support.

^b^Difference between Guiding Pad app and PALS pocket cards groups: *P*=.005 (Fisher exact test).

^c^Difference between Guiding Pad app and PALS pocket cards groups: *P*<.001 (Fisher exact test).

^d^Difference between Guiding Pad app and PALS pocket cards groups: *P*=.001 (Fisher exact test).

Out of 52 opportunities, 1 error in the defibrillation dose (2%) was committed during the whole scenario in group A. This resident delivered a second asynchronous shock at half the recommended energy dose (2 J/kg instead of 4 J/kg). Owing to a discontinuous adherence to the app by switching alternatively with his own CPR experience, he also failed to comply with the algorithm and gave a mistimed 5 mg/kg dose of amiodarone 3 minutes after an unnecessary additional (2 J/kg) second defibrillation attempt. This compares to 8 out of 52 (15%) errors in defibrillation doses during the whole scenario in group B (*P*<.03): 3 at the first defibrillation attempt (doses ranged from 0.6 to 4 J/kg instead of 2 J/kg); 2 at the second attempt (1.0 to 2 J/kg instead of 4 J/kg); and 3 at the third attempt (0.52 to 2 J/kg instead of 4 to 10 J/kg) (see Table 3). Out of 13 residents, 2 in group B (15%) wrongly used synchronized shocks, either at the first, second, or third attempts. In group A, the mean energy dose of the first defibrillation attempt was strictly in accordance with the recommendations, whereas the second, third, and fourth defibrillation attempts deviated from the AHA recommendations by 0.15 J/kg (95% CI of discrepancy: –0.49 to 0.18, *P*=.34), 0.15 J/kg (95% CI of discrepancy: –0.18 to 0.49, *P*=.34), and 0.31 J/kg (95% CI of discrepancy: –0.36 to 0.98, *P*=.34), respectively. In group B, all four mean defibrillation attempts deviated from the AHA recommendations by 0.03 J/kg (95% CI of discrepancy: –0.49 to 0.43; *P*=.89), 0.38 J/kg (95% CI of discrepancy: –0.97 to 0.20; *P*=.17), 0.13 J/kg (95% CI of discrepancy: –1.08 to 0.82; *P*=.77), and 1.45 J/kg (95% CI of discrepancy: 0.44-2.46; *P*=.009), respectively.

In group A, epinephrine drug doses were given according to AHA recommendations. However, in group B, epinephrine was delivered more than 2 minutes, on four occasions, either before the first (three times) or second (one time) shocks, and was once underdosed by 10 times the recommended dose. Regarding amiodarone, among card users, one resident wrongly ordered the drug before the first shock, another after the fourth shock, a third one at 1.4 times the recommended dose, and a resident even ordered a double dose before the fourth shock.

**Figure 11:**
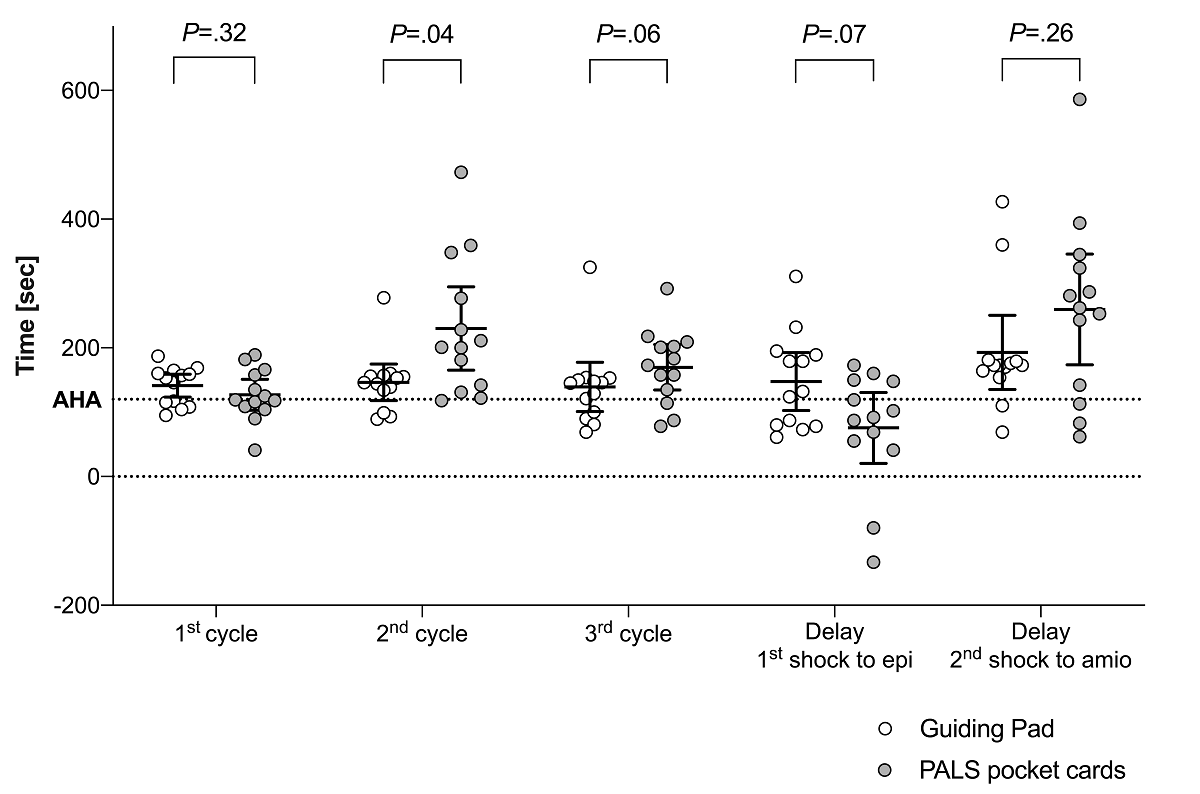
Time spent in seconds by cycles of chest compression and between defibrillation attempts and drug delivery during simulated pulseless ventricular tachycardia (pVT) scenarios. Solid horizontal lines denote mean and 95% CI. White (Guiding Pad app) and grey (pediatric advanced life support [PALS] pocket cards) open circles denote each individual value. The horizontal dashed line denotes the 120-sec American Heart Association (AHA) recommendation for a complete cycle. Delays between the first shock and epinephrine (epi) delivery, and between the second shock and amiodarone (amio) delivery, are expressed as the time to drug delivery minus the time to defibrillation attempt, by resident and by allocation group. A negative time point denotes a drug given before the expected defibrillation attempt.

The hands-on time spent by cycles of chest compression between both groups is summarized in Figure 11. Using the Guiding Pad app, the mean time for the first, second, and third cycles of chest compression between each defibrillation attempt deviated from the AHA recommendations by 21.15 seconds (95% CI of discrepancy: 3.35-38.95; *P*=.02), 26.38 seconds (95% CI of discrepancy: –1.98 to 54.75; *P*=.07), and 19.30 seconds (95% CI of discrepancy: –18.88 to 57.49; *P*=.29), respectively. In group B, the mean time for the first, second, and third cycles of chest compression deviated from the AHA recommendations by 7.08 seconds (95% CI of discrepancy: –17.16 to 31.31; *P*=.54), 110.10 seconds (95% CI of discrepancy: 45.25-174.9; *P*=.003), and 49.85 seconds (95% CI of discrepancy: 14.58-85.11; *P*=.01), respectively. Mean delays between the first shock and epinephrine for the app and pocket card users were 147.7 seconds (95% CI 102.6-192.7) and 75.6 seconds (95% CI 20.5-130.7), respectively (see Figure 11). Mean delays between the second shock and amiodarone for the app and pocket card users were 193.0 seconds (95% CI 135.2-250.8) and 259.6 seconds (95% CI 173.6-345.6), respectively (see Figure 11).

The questionnaire evaluating perceived stress and satisfaction scores was completed and returned by 100% of participants. Participants in groups A and B rated the overall perceived stress before the scenario with mean scores of 5.3 (95% CI 4.0-6.6) and 5.1 (95% CI 3.9-6.3), respectively (*P*=.78). During the scenario, the stress remained contained by the app users (mean score 4.8, 95% CI 3.4-6.2, *P*=.55), whereas it increased significantly for residents relying on the PALS pocket cards (mean score 6.8, 95% CI 5.9-7.8, *P*=.01) compared to app users (*P*=.01). Satisfaction tended to be greater for residents using the app (mean score 7.5, 95% CI 6.5-8.5) compared to those using pocket cards (mean score 5.9, 95% CI 4.4-7.4) (*P*=.07).

## Discussion

### Principal Findings

In this randomized controlled trial, we report a reduced time to all defibrillation attempts and an improved adherence of approximately 70% to all CPR sequences of action outlined by the 2018 AHA pVT guidelines with the mobile app Guiding Pad compared with the PALS pocket reference cards among pediatric residents leading simulated CPR. Of note, this result was observed irrespective of residents’ previous years of experience or prior CPR knowledge. Interindividual variance was also reduced with the app, suggesting a worthwhile benefit of its use by residents with various experience levels. To our knowledge, this is the first study to investigate the benefit of a mobile device app to improve the performance and adherence of pediatric residents to AHA resuscitation guidelines.

Standards of care acknowledge that a prompt defibrillation attempt is an important determinant of survival after cardiac arrest [27]. As outlined by Topijan et al, shorter duration of CPR is associated with higher rates of survival to discharge, supporting the concept of rapid recognition, prompt chest compression, and defibrillation as soon as possible [25]. During the first 15 minutes of CPR, survival and a favorable neurological outcome decrease linearly by 2.1% and 1.2% per minute, respectively [28]. Delays in initiating CPR have a detrimental effect on patient outcome, regardless of the quality of resuscitation [29]. Therefore, the AHA recommends that pulseless patients of any age should receive immediate CPR without delay starting with chest compressions followed by a defibrillation within 180 seconds of a shockable rhythm. In our study, approximately 80% of residents in both allocation groups started compressions within 60 seconds from the onset of pVT. Importantly, mean time from initiation of chest compression to first shock was almost halved when using the app when compared to PALS pocket cards. Among residents using the app, 92% (12/13) defibrillated successfully in 180 seconds or less of pVT onset, whereas 46% (6/13) of PALS pocket card users failed to discharge the defibrillator within 180 seconds. This correlates well with the results of Hunt et al, who observed that despite the availability of AHA recommendations, 66% of pediatric residents failed to start compressions within 60 seconds from the onset of a simulated pVT, 33% never started compressions, only 54% successfully defibrillated within 180 seconds, and 7% never discharged the defibrillator [30]. A more recent study among first-year pediatric residents showed a median time of 50 seconds for the initiation of CPR and 282 seconds to first defibrillation [31]. Most alarmingly, the pVT rhythm in our trial was misidentified by almost 70% of residents holding the PALS reference cards in their hands. This could potentially negatively affect patient outcome as choosing the wrong electrical therapy, drugs, or algorithm in real life might impede the correct management of critically ill children and jeopardize their chance of survival.

Current AHA resuscitation guidelines emphasize 2 minutes of chest compressions between defibrillation attempts as optimal care for persistent pVT or ventricular fibrillation in children [32,33]. In this study, app users deviated less from the AHA, which reached statistical significance for the second and third cycles of chest compression. Moreover, following the first and second shocks and a 2-minute period of five cycles of CPR after each shock, antiarrhythmic drugs should be administered if the patient remains in cardiac arrest, with the aim of increasing defibrillation success with subsequent defibrillation attempts [32]. In this trial, both groups accurately administered epinephrine and amiodarone drug doses, with the exception of a 100-times underdosed epinephrine and a 1.4-times overdosed amiodarone dose in group B. On average, app users correctly respected a complete 2-minute cycle of chest compression-ventilation before administering epinephrine after the first shock. Conversely, and contrary to current AHA guidelines, pocket card users administered the drug too close to the first shock, possibly explaining the absence of a significant time difference to epinephrine administration between both groups, despite a significantly longer delay to deliver the first shock in group B. Due to further delays, amiodarone was delivered significantly later by more than 2 minutes among residents not using the app.

Prompt defibrillation is crucial for the termination of ventricular fibrillation or pVT in order to achieve ROSC [33]. The AHA 2018 guidelines recommend treating pVT or ventricular fibrillation in children with an initial dose of 2 J/kg [23]. For subsequent shocks, a dose of 4 J/kg is recommended, although higher energy levels may be considered up to an adult dose, if not exceeding 10 J/kg. In this trial, residents using the PALS pocket cards were more prone to deviate from defibrillation doses than those using the app. In a total of 52 defibrillation attempts, they deviated in 36% of cases. These deviations were reduced to 6% when using the app. It would be interesting in further studies to determine whether this would translate into fewer deviations in shock doses in real life.

While the app in this study offered better adherence to AHA resuscitation recommendations than conventional PALS pocket cards, we also found that it provided a relative advantage when compared to the Google Glass-based app dedicated to the same purpose [17]. The in-built small size of the screen was indeed a limiting factor reported by residents wearing the glasses, by hindering full display of algorithms. Usability issues were also observed with inopportune and time-consuming back-and-forth navigations throughout the algorithms. In this study, displaying the entire algorithm on the larger screen size of a tablet and paralleling stepwise patient-centered care guidance appeared to improve adherence to AHA guidelines and speed up skills, thus allowing residents to better manage simulated CPR. It would be interesting in further studies to assess this assumption with certified emergency physicians or paramedics in simulated and real-life in- or out-of-hospital environments. Given the evidence regarding the observed deviation from recommended resuscitation procedures, it might be also advisable to assess the educational impact of this app for the upstream training of rescuers’ p-IHCA technical skills in further studies.

### Limitations

Our study has some limitations. First, it was conducted during a resuscitation simulation–based scenario rather than tested in real-life situations. However, high-fidelity simulation is an essential method to teach resuscitation skills and technologies that cannot be practiced during real CPR, as the diversity among patients and their diseases makes such studies difficult to standardize in critical situations. The low occurrence of p-IHCA also limits the implementation of randomized trials in real life [34]. Moreover, standardizing the scenario and the environment helped to avoid effect modifiers by limiting the influence of undesired variables on the outcomes. Realism was achieved as reflected by the stress level experienced by participants, who considered the simulation to be as stressful as real CPR situations. Second, the 5-minute app training was dispensed just before the scenario. In real life, the interval between training and actual use would probably be months. However, training with the app months before the study would have unblinded participants to its purpose and could have created a preparation bias. Third, the sample size limited stratified analyses to estimate the impact of PALS certification on the outcomes, but a recent study observed that improved adherence to AHA recommendations was not directly associated with PALS-trained providers [7]. Finally, we acknowledge that our findings might not be generalizable to providers with extensive CPR experience, such as pediatric emergency physicians. As only residents were assessed in this trial, further studies would be valuable to assess this assumption.

### Conclusions

A PALS-based mobile app designed for tablets to interactively support residents during pediatric CPR contributed to a shorter time to first and subsequent defibrillation attempts, fewer medication and defibrillation dose errors, as well as a better adherence to AHA recommendations, compared with the conventional PALS pocket reference cards. Taken together, our results suggest that residents are not accurately following AHA recommendations during pediatric CPR when only supported by PALS pocket cards. A next step would be to determine, in real-life studies, whether this mobile app might benefit patients by improving the adherence and performance of residents to meet AHA resuscitation requirements in clinical practice.

## Appendix

Multimedia Appendix 1 CONSORT-eHEALTH V1.6.2.

Multimedia Appendix 2 The pulseless ventricular tachycardia (pVT) resuscitation scenario.

Multimedia Appendix 3 Questionnaire for secondary outcomes, using 10-point Likert scales.

Multimedia Appendix 4 Interrater agreement on outcome analyses.

Multimedia Appendix 5 Bland and Altman analysis and intraclass correlation coefficient (ICC) on outcome analyses.

Multimedia Appendix 6 Bland and Altman analysis of pulseless ventricular tachycardia (pVT) algorithm review.

Multimedia Appendix 7 Association between time to defibrillation attempts and number of prior cardiopulmonary resuscitation (CPR) attempts.

Multimedia Appendix 8 Association between time to chest compression or drug delivery and number of prior cardiopulmonary resuscitation (CPR) attempts.

## Abbreviations

AHA: American Heart Association
BLS: basic life support
CONSORT-EHEALTH: Consolidated Standards of Reporting Trials of Electronic and Mobile Health Applications and Online TeleHealth
CPR: cardiopulmonary resuscitation
PALS: pediatric advanced life support
PED: pediatric emergency department
p-IHCA: pediatric in-hospital cardiac arrest
p-OHCA: pediatric out-of-hospital cardiac arrest
pVT: pulseless ventricular tachycardia
ROSC: return of spontaneous circulation
