# Supplementary material for: The Impact of a Tablet App on Adherence to American Heart Association Guidelines During Simulated Pediatric Cardiopulmonary Resuscitation: Randomized Controlled Trial
Source: J Med Internet Res. 2020 May 27;22(5):e17792. doi: 10.2196/17792 (PMC7287744; doi:10.2196/17792)
Supplement: Multimedia Appendix 1 [file jmir_v22i5e17792_app1.pdf]

CONSORT-EHEALTH Checklist V1.6.2 Report

(based on CONSORT-EHEALTH V1.6), available at [http://tinyurl.com/consort-ehealth-v1-6].

Date completed

13.01.20 18:00

by

Johan N. Siebert

TITLE

"Assessing the Impact of a Mobile Device Tablet App to Increase Adherence to American Heart Association Guidelines During Pediatric Cardiopulmonary Resuscitation: Randomized Controlled Trial"

1a) Identification as a randomised trial in the title

"Randomised, controlled trial "

1a-i) Identify the mode of delivery in the title

"Mobile Device Tablet App"

1a-ii) Non-web-based components or important co-interventions in title

NA

1a-iii) Primary condition or target group in the title

"During Pediatric Cardiopulmonary Resuscitation"

ABSTRACT

1b-i) Key features/functionalities/components of the intervention and comparator in the METHODS section of the ABSTRACT

"Summary

Background: Evidence-based best practices are the cornerstone to guide optimal cardiopulmonary arrest resuscitation care. Adherence to the American Heart Association (AHA) guidelines for cardiopulmonary resuscitation (CPR) optimizes the management of critically ill patients and increases their chances of survival after cardiac arrest. However, a suboptimal quality of CPR remains common in pediatrics, with only approximately 38% of children surviving hospital discharge after in-hospital cardiac arrest and only 6-20% after out-of-hospital cardiac arrest.

Objective: We investigated whether a mobile app developed as a guide to support and drive CPR providers in real-time through interactive pediatric advanced life support (PALS) algorithms would increase adherence to AHA guidelines and reduce the time to initiation of critical life-saving maneuvers compared to the use of PALS pocket reference cards.

Methods: This study was a randomized controlled trial conducted during a simulation-based pediatric cardiac arrest scenario-pulseless ventricular tachycardia (pVT). Twenty-six pediatric residents were randomized into two groups. The primary outcome was the elapsed time in seconds in each allocation group from the onset of pVT to the first defibrillation attempt. Secondary outcomes were time elapsed to 1) initiation of chest compression, 2) subsequent defibrillation attempts, and 3) administration of drugs, including the time intervals between defibrillation attempts and drug doses, shock doses, and the number of shocks. All outcomes were assessed for deviation from AHA guidelines.

Results: Mean time to the first defibrillation attempt (121.4 sec [95% CI 105.3-137) was significantly reduced using the app compared to PALS pocket cards (211.5 sec [95% CI 162.5-260.6], P <.001). With the app, 11/13 (84.6%) residents initiated chest compressions within 60 sec from the onset of pVT and 12/13 (92.3%) successfully defibrillated within 180 sec. Time to all other defibrillation attempts were reduced with the app. Adherence to the 2018 AHA pVT algorithm improved by approximately 70% (P =.001) using the app following all CPR sequences of action in a stepwise fashion until return of spontaneous circulation. The pVT rhythm was recognized correctly in 51/52 (98.1%) opportunities using the app compared to only 19/52 (36.5%) among those using PALS cards (P<.001). Time to epinephrine injection was similar. Among a total of 78 opportunities, wrong shock or drug doses occurred in 14.1% (11/78) of cases among those using the cards. These errors were reduced by 12.8% (1/78, P =.0046) using the app.

Conclusion: Use of the mobile app was associated with a shorter time to first and subsequent defibrillation attempts, lesser medication and defibrillation dose errors, and improved adherence to AHA recommendations compared with the use of PALS pocket cards.

Trial Registration: Registration was not required as the purpose of the study was to examine the effect of the intervention on healthcare providers.

1b-ii) Level of human involvement in the METHODS section of the ABSTRACT

See abstract content in "subitem 1b-i" section above

1b-iii) Open vs. closed, web-based (self-assessment) vs. face-to-face assessments in the METHODS section of the ABSTRACT

"a randomized controlled trial conducted during a simulation-based pediatric cardiac arrest scenario-pulseless ventricular tachycardia (pVT)"

1b-iv) RESULTS section in abstract must contain use data

See abstract content in "subitem 1b-i" section above

1b-v) CONCLUSIONS/DISCUSSION in abstract for negative trials

"Use of the mobile app was associated with a shorter time to first and subsequent defibrillation attempts, lesser medication and defibrillation dose errors, and improved adherence to AHA recommendations compared with the use of PALS pocket cards"

INTRODUCTION

2a-i) Problem and the type of system/solution

"Pediatric cardiac arrest (CA) is a high-risk low-frequency event associated with death or severe neurological sequelae in survivors. It requires immediate recognition and care by skilled health providers. Recent studies show that in-hospital pediatric CA (p-IHCA) affects 7100 to 8300 children per year in the USA [1], of which 14% occur in pediatric emergency departments (PEDs) [2]. Pediatric out-of-hospital CA (p-OHCA) accounts for a further 7037 children brought to USA PEDs by emergency medical services each year [3]. Despite advances in resuscitation science and survival improvement over the last decades, survival remains low, with only approximately 38% of children surviving to hospital discharge after IHCA, and 6% to 20% after OHCA [3, 4]. Evidence-based best practices are the cornerstone for the guidance of optimal cardiopulmonary arrest resuscitation care. High-quality cardiopulmonary resuscitation (CPR) according to the American Heart Association (AHA) life support guidelines is associated with a successful return of spontaneous circulation (ROSC), improved survival after hospital discharge, and good neurological outcomes [5]. Deviation from recommended procedures are associated with a reduced likelihood of survival from CA [6].

While adherence to AHA guidelines in emergency departments has been described in adults, there are limited data in PEDs [7]. Reference tools for pediatric emergency physicians to handle pediatric CPR according to AHA guidelines are available on reference pocket cards. Unfortunately, healthcare providers frequently do not perform resuscitation according to guidelines despite cognitive aids [8] and AHA life-support training courses, such as basic life support (BLS) and pediatric advanced life support (PALS). Suboptimal quality of CPR is still commonly encountered for both adult and pediatric patients [9].

New resuscitation strategies using information technologies and devices aiming to improve both in- and out-of-hospital CPR have been assessed to ensure adherence to AHA guidelines [10-16]. Nevertheless, research in this area remains scarce, especially in pediatrics, and studies assessing the impact of information technology on p-IHCA management and improved pediatric CPR outcomes are necessary. In a previous randomized trial, we found that adherence to PALS algorithms when adapted on Google glasses was improved with a significant reduction of errors and deviations in defibrillation doses by 53% when compared to the use of pocket reference cards [17]. However, time to the first defibrillation attempt and adherence to AHA guidelines to other critical resuscitation endpoints in terms of time and drug dose delivery were not improved using the glasses. The complexity of interacting while wearing glasses, as well as the limits of the system to situate the current action in the whole resuscitation process and their small size were major limitations to their potential use in p-IHCA. Thus, we have developed a new mobile application (the “Guiding Pad” app) from the ground up and dedicated to tablets. It is intended as a guide to support and drive CPR providers in real-time conditions through interactive PALS algorithms enhanced with patient-centered cognitive aids.

2a-ii) Scientific background, rationale: What is known about the (type of) system

See "subitem 2a-i"

METHODS

3a) CONSORT: Description of trial design (such as parallel, factorial) including allocation ratio

"We conducted a prospective, randomized controlled trial in a tertiary PED (>33,000 consultations/year) with two parallel groups of voluntary pediatric residents. We compared time to the first defibrillation attempt and other critical resuscitation endpoints using a mobile device tablet app (“Guiding Pad”, group A) or AHA PALS conventional pocket reference cards (group B) during a standardized simulation-based pediatric CA scenario."

3b) CONSORT: Important changes to methods after trial commencement (such as eligibility criteria), with reasons

Not applicable. Methods were not changed after trial commencement.

3b-i) Bug fixes, Downtimes, Content Changes

"No changes were made to the app during the study."

4a) CONSORT: Eligibility criteria for participants

"Any physician performing a residency in pediatrics was eligible. Shift-working residents were randomly recruited on the day of the study using an alphabetical list to avoid preparation bias. Included participants benefited from a standardized 5-min introduction course on the use of the tablet app. As BLS training is a requirement for residents at our institution, all participants had previously completed this course prior to study entry. Participation to a simulation in the past month was an exclusion criterion to avoid a recent training effect."

4a-i) Computer / Internet literacy

Not applicable

4a-ii) Open vs. closed, web-based vs. face-to-face assessments:

"Shift-working residents were randomly recruited on the day of the study using an alphabetical list to avoid preparation bias"

4a-iii) Information giving during recruitment

"Written informed consent was obtained from each participant after full information disclosure prior to study participation. Blinding to the purpose of the study was maintained during recruitment to minimize preparation bias. Allocation concealment was managed with the software and was not released until the participant started the scenario"

4b) CONSORT: Settings and locations where the data were collected

"We conducted a prospective, randomized controlled trial in a tertiary PED (>33,000 consultations/year)"

"We compared time to the first defibrillation attempt and other critical resuscitation endpoints using a mobile device tablet app ("Guiding Pad", group A) or AHA PALS conventional pocket reference cards (group B) during a standardized simulation-based pediatric CA scenario."

4b-i) Report if outcomes were (self-)assessed through online questionnaires

Outcomes were not (self-)assessed through online questionnaires. Demographic data were assessed through questionnaires directly given to the participants on the day of participation.

4b-ii) Report how institutional affiliations are displayed

"Shift-working residents were randomly recruited on the day of the study using an alphabetical list to avoid preparation bias".
During enrollment, the residents were informed that the Guidng Pad app was developed by the Geneva University Hospitals.

5) CONSORT: Describe the interventions for each group with sufficient details to allow replication, including how and when they were actually administered

5-i) Mention names, credential, affiliations of the developers, sponsors, and owners

"Geneva University Hospitals are the owner of the app "Guiding Pad," which is not available at the time of submission on the Google Play Store and Apple App Store. All authors declare no conflicts of interest. The present trial had financial support from the private foundation of Geneva University Hospitals (fund no. QS2-25). The funder of the study had no role in study design, data collection, data analysis, data interpretation, or writing of the report."

5-ii) Describe the history/development process

"Unlike adults, CA in children without prior cardiac diseases is mainly due to asystole (40%) and pulseless electrical activity (24%) [2]. As ventricular fibrillation and pVT, namely shockable rhythms, have been identified in 27% of p-IHCA [20], we decided to use the pVT algorithm as we considered that it would offer a greater opportunity to assess the multiple-step resuscitative skills set out in the AHA guidelines. . The app was developed at Geneva University Hospitals using Angular version 8, a development framework created by Google to build mobile and web apps. The AHA PALS algorithms were adapted for tablet devices following a user-centered and ergonomic-driven approach by computer scientists, senior pediatric emergency physicians and ergonomists."

5-iii) Revisions and updating

"No changes were made to the app during the study"

5-iv) Quality assurance methods

"The trial was conducted according to the principles of the Declaration of Helsinki and Good Clinical Practice guidelines, and in accordance with the Consolidated Standards of Reporting Trials of Electronic and Mobile Health Applications and Online TeleHealth guidelines (Multimedia Appendix 1) [18], including also Reporting Guidelines for Health Care Simulation Research [19]."

The study "was conducted during a resuscitation simulation-based scenario rather than tested in real-life situations. However, high-fidelity simulation is an essential method to teach resuscitation skills and technologies that cannot be practiced during real CPR as the diversity among patients and their diseases makes such studies difficult to standardize in critical situations. The low occurrence of p-IHCA also limits the implementation of randomized trials in real life [33]. Moreover, standardizing the scenario and the environment helped to avoid effect modifiers by limiting the influence of undesired variables on the outcomes."

"[...] each participant allocated to the “Guiding Pad” group (group A) received a standardized 5-min training session on how to use the app".

"The scenario was standardized to strictly follow the 2018 AHA pediatric pVT algorithm (Figure 3) and performed on the same high-fidelity manikin already primed with vital signs appropriate for the scenario (Multimedia Appendix 2). It was conducted in situ in the PED shock room to increase realism, thus allowing participants to make use of real resources in the actual environment where they were expected to handle CA. All participants in group B were offered the possibility to hold PALS pocket reference cards in their hands throughout the entire scenario. Whether they referred to it or not was left to their discretion, similar to real life settings. No interactions occurred between participants and investigators. The simulation involved a resuscitation team comprising three study team members, i.e., a PED registered nurse and two medical students to assist with resuscitation through drug preparation, chest compression and bag-valve-mask ventilation, and the participating resident. Study team members had no role in decision making to achieve ROSC. A PALS instructor (a pediatric emergency physician) who was not a member of the resuscitation team operated the simulator. To be consistent with the 2018 AHA pediatric CA algorithm [22] and to standardize the scenario, defibrillation doses of 2 Joules per kg for the first attempt, and 4 Joules per kg for the subsequent second, third and fourth attempts were expected (Figure 3)".

"All actions (i.e. primary and secondary outcomes) performed by the resident during the scenario were independently recorded by two trained investigators blinded to each other’s records during the simulation, thus allowing an accurate assessment of timing and sequencing of actions, and to avoid assessment bias. In the case of disagreement, a third independent evaluator helped reach a consensus"

"All actions performed by the provider are automatically saved in log files to preserve information that can be retrieved at any time for debriefing or medicolegal purposes."

5-v) Ensure replicability by publishing the source code, and/or providing screenshots/screen-capture video, and/or providing flowcharts of the algorithms used

The full resuscitation scenario used in this trial is detailed in the Methods section of the study. See also the supplementary material 2.

5-vi) Digital preservation

The "Guiding Pad" app is owned by the Geneva University Hospitals and secured on local servers. The app was assessed on an iPad (Apple) interfaced with iOS.

5-vii) Access

"On the day of participation, each resident completed an anonymous survey on basic demographic information, professional length of clinical experience, and PALS training. After random allocation, each participant received a standardized 5-min training session on how to use the app. Participants were then asked to perform a 15-min highly realistic scripted CPR scenario on a high-fidelity manikin (SimJunior; Laerdal Medical, Stavanger, Norway)."

See "subitem 5-vi" above for digital access.

5-viii) Mode of delivery, features/functionalities/components of the intervention and comparator, and the theoretical framework

Please see "Methods" section for complete description of the Control and Intervention arms.

5-ix) Describe use parameters

Not applicable as the app was used once on the day of study participation.

5-x) Clarify the level of human involvement

"The simulation involved a resuscitation team comprising three study team members, i.e., a PED registered nurse and two medical students to assist with resuscitation through drug preparation, chest compression and bag-valve-mask ventilation, and the participating resident. Study team members had no role in decision making to achieve ROSC. A PALS instructor (a pediatric emergency physician) who was not a member of the resuscitation team operated the simulator."

This point was described in details in the "Procedures" section.

5-xi) Report any prompts/reminders used

Not applicable as the app was used once on the day of study participation.

5-xii) Describe any co-interventions (incl. training/support)

"After random allocation, each participant received a standardized 5-min training session on how to use the app."

"All participants in group B were offered the possibility to hold PALS pocket reference cards in their hands throughout the entire scenario. Whether they referred to it or not was left to their discretion, similar to real life settings. No interactions occurred between participants and investigators."

"Participants were informed of the results after completion of the study."

6a) CONSORT: Completely defined pre-specified primary and secondary outcome measures, including how and when they were assessed

"The primary outcome was the delay (in seconds) in each allocation group from the end of the clinical statement given by the study investigator to the first defibrillation attempt, as the expected survival advantage from early CPR can be significantly affected by a subsequent delay in defibrillation [23, 24]. Secondary outcomes were the delay (in seconds) to initiation of chest compression, time to subsequent defibrillation attempts, time to administration of epinephrine and amiodarone, time interval (in seconds) between defibrillation attempts, drug doses, shock doses and number of shocks, and perceived stress and satisfaction scores after completion of the scenario, as measured by a questionnaire using 10-point Likert scales (Multimedia Appendix 3). AHA recommends five cycles of chest compression (approximately 2 min) between each defibrillation attempt. The time spent by participants to perform chest compressions by compression cycles was defined as the hands-on time and measured in seconds with a chronometer. All these outcomes were assessed for deviation from AHA guidelines."

"All actions (i.e. primary and secondary outcomes) performed by the resident during the scenario were independently recorded by two trained investigators blinded to each other's records during the simulation, thus allowing an accurate assessment of timing and sequencing of actions, and to avoid assessment bias. In the case of disagreement, a third independent evaluator helped reach a consensus. Data were manually retrieved and entered into a Microsoft Excel spreadsheet version 16 (Microsoft Corp.). Unaccomplished actions were left blank and time was not assigned. Residents' privacy was preserved. Only the study investigators had access to the data."

6a-i) Online questionnaires: describe if they were validated for online use and apply CHERRIES items to describe how the questionnaires were designed/deployed

Not applicable

6a-ii) Describe whether and how "use" (including intensity of use/dosage) was defined/measured/monitored

Please refer to point 6a above.

6a-iii) Describe whether, how, and when qualitative feedback from participants was obtained

"No participant was asked for advice on the interpretation or writing of the study results. Participants were informed of the results after completion of the study."

6b) CONSORT: Any changes to trial outcomes after the trial commenced, with reasons

Due to the nature of our simulation based-study, we made no changes to the trial.

7a) CONSORT: How sample size was determined

7a-i) Describe whether and how expected attrition was taken into account when calculating the sample size

"Power calculations were based on the detection of a 30-sec decrease in time to the first defibrillation attempt between the two independent groups. A previous study has shown a mean time to first defibrillation of 92 sec with a standard deviation (SD) of 23 sec [25]. Assuming a similar SD in each group in our study, 10 participants per group had to be recruited to provide the trial with 80% power at a two-sided alpha level of .05. To prevent a potential loss of power due to misspecification of assumptions, 13 participants were recruited per group, giving a total sample size of 26 participants."

7b) CONSORT: When applicable, explanation of any interim analyses and stopping guidelines

Due to the nature of our simulation based-study, no interim analyses nor stopping guidelines were required.

8a) CONSORT: Method used to generate the random allocation sequence

"Residents were randomized using a single constant 1:1 allocation ratio determined with a web-based software (www.sealedenvelopes.com)."

8b) CONSORT: Type of randomisation; details of any restriction (such as blocking and block size)

See "subitem 8a"

9) CONSORT: Mechanism used to implement the random allocation sequence (such as sequentially numbered containers), describing any steps taken to conceal the sequence until interventions were assigned

"Residents were randomized using a single constant 1:1 allocation ratio determined with a web-based software (www.sealedenvelopes.com) [...] Allocation concealment was managed with the software and was not released until the participant started the scenario."

10) CONSORT: Who generated the random allocation sequence, who enrolled participants, and who assigned participants to interventions

See "subitem 8a".

As mentioned above "Shift-working residents were randomly recruited on the day of the study using an alphabetical list to avoid preparation bias". Participants were assigned by the study investigators to their respective study arms on the day of participation according to the randomisation list.

11a) CONSORT: Blinding - If done, who was blinded after assignment to interventions (for example, participants, care providers, those assessing outcomes) and how

11a-i) Specify who was blinded, and who wasn't

"Blinding to the purpose of the study was maintained during recruitment to minimize preparation bias."

The data analyst (JS) was not blinded to treatment allocation after completion of the scenario.

11a-ii) Discuss e.g., whether participants knew which intervention was the "intervention of interest" and which one was the "comparator"

"Allocation concealment was managed with the software and was not released until the participant started the scenario."

11b) CONSORT: If relevant, description of the similarity of interventions

"Participants were then asked to perform a 15-min highly realistic scripted CPR scenario on a high-fidelity manikin (SimJunior; Laerdal Medical, Stavanger, Norway). The scenario was standardized to strictly follow the 2018 AHA pediatric pVT algorithm (Figure 3) and performed on the same high-fidelity manikin already primed with vital signs appropriate for the scenario (Multimedia Appendix 2). It was conducted in situ in the PED shock room to increase realism, thus allowing participants to make use of real resources in the actual environment where they were expected to handle CA. All participants in group B were offered the possibility to hold PALS pocket reference cards in their hands throughout the entire scenario. Whether they referred to it or not was left to their discretion, similar to real life settings."

12a) CONSORT: Statistical methods used to compare groups for primary and secondary outcomes

"Power calculations were based on the detection of a 30-sec decrease in time to the first defibrillation attempt between the two independent groups. A previous study has shown a mean time to first defibrillation of 92 sec with a standard deviation (SD) of 23 sec [25]. Assuming a similar SD in each group in our study, 10 participants per group had to be recruited to provide the trial with 80% power at a two-sided alpha level of .05. To prevent a potential loss of power due to misspecification of assumptions, 13 participants were recruited per group, giving a total sample size of 26 participants.

For the primary analysis, we first evaluated the time elapsed between the onset of pVT and first defibrillation attempt. The Shapiro-Wilks test was used for normality analysis of the parameters. As most of the continuous variables were normally distributed, means and SDs with their 95% confidence interval (CI) were reported. Non-normally distributed variables were analyzed using a Mann-Whitney test. Frequencies were reported as percentages. T tests were used to compare independent groups. No paired data were compared. Kaplan–Meier curves for time elapsed between the onset of pVT and first defibrillation attempt were estimated and compared using the log-rank (Mantel-Cox) test for bivariate survival analysis.

For the secondary analysis, we evaluated the time elapsed between the onset of pVT to subsequent defibrillation attempts and the delivery of both drugs. For normally distributed variables, means and SDs with 95% CI were reported. Non-normally distributed variables were analyzed using a Mann-Whitney test. Frequencies were reported as percentages. T tests were used to compare independent groups. No paired data were compared. Kaplan–Meier curves for time elapsed between the onset of pVT and subsequent defibrillation attempts and delivery of both drugs were also estimated and compared using the log-rank (Mantel-Cox) test for bivariate survival analysis. Errors in cycles of chest compression-ventilation were measured as the deviation in percent from the experimental time spent in seconds compared to the 2-min duration recommended by AHA. Wrong defibrillation or drug doses were measured as a deviation from the amount of energy delivered in Joules or drug doses in milliliters compared to AHA recommendations. A χ^2 test was used to assess the relationship between absolute errors in defibrillation and drug doses expressed as categorical variables. Wrong defibrillation mode was also measured. Absolute deviations were also analyzed. The mean (SD) difference in deviation obtained with each method was reported with a 95% CI. A t test for unpaired data was used to compare interventions. Mean differences were reported by randomized group. Univariate linear regressions analysis with 95% CI were performed to assess whether time to initiation of chest compression, defibrillation attempts and drug delivery were associated with the number of postgraduate years or prior resuscitation experience as a provider in real-life and simulated environments. Means and SDs were determined for the perceived stress and satisfaction scores of individuals derived from the Likert-scale questionnaire and reported with descriptive statistics. A P value less than .05 was considered significant.

Interrater reliability was assessed by two observers who independently evaluated each resident's performance. Interrater reliability scores were calculated using Cohen's kappa coefficient for the shock and drug dose errors. As the remaining outcomes were continuous variables, the Bland-Altman method was used to plot the difference of values reported by both observers against the mean value for each outcome. The limits of agreement were assessed by the interval of ± 1.96 SD of the measurement differences either side of the mean difference. The null hypothesis that there was no difference on average between both reviewers was tested using a t-test. The mean difference was reported with its 95% CI. Additionally, the intraclass correlation coefficients for times to each critical endpoint were assessed, assuming that raters

12a-i) Imputation techniques to deal with attrition / missing values

Due to the design of the study, no imputation was planned.

12b) CONSORT: Methods for additional analyses, such as subgroup analyses and adjusted analyses

See "subitem 12a". No additional analysis were conducted.

RESULTS

13a) CONSORT: For each group, the numbers of participants who were randomly assigned, received intended treatment, and were analysed for the primary outcome

"[...] 26 pediatric residents were assessed for eligibility and randomly assigned to either the “Guiding Pad” app group A (n=13) or the PALS pocket card group B (n=13), without any dropouts or missing data (Figure 5). Baseline characteristics were balanced in the two groups (Table 1)."

13b) CONSORT: For each group, losses and exclusions after randomisation, together with reasons

"[…] without any dropouts or missing data (Figure 5). "

13b-i) Attrition diagram

See Figure 5 in the manuscript

14a) CONSORT: Dates defining the periods of recruitment and follow-up

"From 30 August to 17 October 2019"

14a-i) Indicate if critical “secular events” fell into the study period

Not applicable

14b) CONSORT: Why the trial ended or was stopped (early)

Not applicable

15) CONSORT: A table showing baseline demographic and clinical characteristics for each group

See Table 1 in the manuscript

15-i) Report demographics associated with digital divide issues

See Table 1 in the manuscript

16a) CONSORT: For each group, number of participants (denominator) included in each analysis and whether the analysis was by original assigned groups

16-i) Report multiple “denominators” and provide definitions

See Figure 1 in the manuscript.

16-ii) Primary analysis should be intent-to-treat

Not applicable. Due to nature of the study, no deviations from protocol occurred. Primary analysis were therefore not based on an intent-to-treat.

17a) CONSORT: For each primary and secondary outcome, results for each group, and the estimated effect size and its precision (such as 95% confidence interval)

"Baseline characteristics were balanced in the two groups (Table 1). We observed perfect interrater agreement for the scoring of the pVT scenario (Table S1, Figure S1, and Table S2)."

Time to Resuscitation Critical Endpoint:

Using the “Guiding Pad”, 11/13 (84.6%) residents initiated chest compressions within 60 sec of the onset of pVT (69.2% within 30 sec), and 12/13 (92.3%) successfully defibrillated within 180 sec (Figure 6). Mean time elapsed between the onset of pVT and first defibrillation attempt was 121.4 sec (SD 26.7). With PALS pocket cards, 10/13 (76.9%) residents started compressions within 60 sec, one resident (7.7%) started compressions 277 sec after onset of pVT, and 6/13 (46.2%) failed to discharge the defibrillator within 180 sec. Mean time from initiation of chest compression to the first shock was significantly reduced for residents using the app (89.3 sec) than those using the PALS pocket cards (163 sec; P=.0016). Mean times to other resuscitation critical endpoints are summarized in Table 2. All defibrillation attempts, as well as amiodarone administration, were delivered significantly earlier in group A than in group B. However, the app was unable to speed up the delay before intraosseous access and epinephrine delivery (Table 2 and Figure 7). We sought to analyze in both groups the difference in mean time to first defibrillation attempts between residents with or without previous defibrillation experience in either real-world or simulated environments, but with our small sample size we did not find any difference (with the app: 124.1 vs 112.3 sec, P = .53; without the app: 268.8 vs 186.1 sec, P = .090). At the time of the study, 24/26 (92.3%) participants were residents with more than one year of pediatric training (postgraduate years). In a simple linear regression model, using the app was associated with a significant or borderline significant reduction in time to defibrillation attempts, regardless of the postgraduate years, and less scattered delays around the mean defibrillation time than when using the pocket cards (Figure 8). In group B, time to defibrillation attempts was inversely associated with the number of postgraduate years. In both groups, we observed no correlation between the time to initiation of chest compression or time to drug delivery and postgraduate years (Figure 9). Moreover, we observed no relationship between previous CPR experience expressed as the number of prior CPR attempts on either a patient or a manikin and times to initiation of CPR, defibrillation attempts or drug delivery (Figures S2 and S3).

Errors and Deviations from the AHA pVT Algorithm and Errors:

Errors and deviations from the AHA pVT algorithm are summarized in Table 3 and supplementary Table 1. The entire pVT algorithm was followed correctly in a stepwise fashion until ROSC by 12/13 (92.3%) residents in group A and only 3/13 (23.1%) in group B (P = .001) (Table 3). Importantly, the pVT rhythm was recognized correctly in 51/52 opportunities (98.1%) by residents using the app, but in only 19/52 (36.5%) of those using the pocket card (P < .001). Out of 52 opportunities, one error in the defibrillation dose (1.9%) was committed during the whole scenario in group A. This resident delivered a second asynchronous shock at half the recommended energy dose (2 J/kg instead of 4 J/kg). Owing to a discontinuous adherence to the app by switching alternatively with his own CPR experience, he also failed to comply with the algorithm and gave a mistimed 5 mg/kg dose of amiodarone 3 min after an unnecessary additional (2 J/kg) second defibrillation attempt. This compares to 8/52 (15.4%) errors in defibrillation doses during the whole scenario in group B (P < .031); three at the first defibrillation attempt (doses ranged from 0.6 to 4 J/kg instead of 2 J/kg); two at the second attempt (1.0 to 2 J/kg instead of 4 J/kg); and three at the third attempt (0.52 to 2 J/kg instead of 4 to 10 J/kg) (Table 3). Out of 13, two residents group B (15.4%) wrongly used synchronized shocks (either at the first, second or third attempts). In group A, the mean energy dose of the first defibrillation attempt was strictly in accordance with the recommendations, whereas the second, third and fourth defibrillation attempts deviated from the AHA recommendations by 0.15 J/kg (95% CI of discrepancy: -0.49 to 0.18; P = .34), 0.15 J/kg (95% CI of discrepancy: -0.18 to 0.49; P = .34), and 0.31 J/kg (95% CI of discrepancy: -0.36 to 0.98; P = .34), respectively. In group B, all four mean defibrillation attempts deviated from the AHA recommendations by 0.03 J/kg (95% CI of discrepancy: -0.49 to 0.43; P=.89), 0.38 J/kg (95% CI of discrepancy: -0.97 to 0.20; P = .17), 0.13 J/kg (95% CI of discrepancy: -1.08 to 0.82; P = .77), and 1.45 J/kg (95% CI of discrepancy: 0.44 to 2.46; P = .0088), respectively. In group A, epinephrine drug doses were given accordingly to AHA recommendations. However, in group B, epinephrine was delivered more than 2 min on four occasions, either before the first (three times) or second shocks, and was once underdosed by 10 times the recommended dose. Regarding amiodarone, among card users, one resident wrongly ordered the drug before the first shock, another after the fourth shock, a third one at 1.4 times the recommended dose, and a resident even ordered a double dose before the fourth shock.

The hands-on time spent by cycles of chest compression between both groups is summarized in Figure 10. Using the “Guiding Pad”, the mean time for the first, second and third cycles of chest compression between each defibrillation attempts deviated from the AHA recommendations by 21.15 sec (95% CI of discrepancy: 3.35 to 38.95; P = .024), 26.38 sec (95% CI of discrepancy: -1.98 to 54.75; P = .066), and 19.3 sec (95% CI of discrepancy: -18.88 to 57.49; P = .29), respectively. In group B, the mean time for the first, second and third cycles of chest compression deviated from the AHA recommendations by 7.08 sec (95% CI of discrepancy: (-17.16 to 31.31; P = .54), 110.1 sec (95% CI of discrepancy: 45.25 to 174.9; P = .0030), and 49.85 sec (95% CI of discrepancy: 14.58 to 85.11; P = .0095), respectively. Mean delays between the first shock and epinephrine for the app and pocket card users were 147.7 sec [95% CI: 102.6 to 192.7] and 75.6 sec [20.5 to 130.7], respectively (Figure 10). Mean delays between the second shock and amiodarone for the app and pocket card users were 193.0 sec [135.2 to 250.8] and 259.6 sec [173.6 to 345.6], respectively (Figure 10).

The questionnaire evaluating perceived stress and satisfaction scores was completed and returned by 100% of participants. Participants in groups A and B rated the overall perceived stress before the scenario to be 5.3 (95% CI 4.0 to 6.6) and 5.1 (95% CI 3.9 to 6.3), respectively (P = .78). During the scenario, the stress was rated to remain contained by the app users (4.8 [95% CI 3.4 to 6.2], P = .55), whereas it increased significantly for residents relying on the PALS pocket cards (mean 6.8 [95% CI 5.9 to 7.8], P = .014) compared to app users (P=.012). Satisfaction tended to be greater using the app (7.5 [95% CI 6.5 to 8.5] vs 5.9 [95% CI 4.4 to 7.4], respectively, P = .07)."

17a-i) Presentation of process outcomes such as metrics of use and intensity of use

See "subitem 17a"

17b) CONSORT: For binary outcomes, presentation of both absolute and relative effect sizes is recommended

See "subitem 17a"

18) CONSORT: Results of any other analyses performed, including subgroup analyses and adjusted analyses, distinguishing pre-specified from exploratory

See "subitem 17a"

18-i) Subgroup analysis of comparing only users

See "subitem 17a"

19) CONSORT: All important harms or unintended effects in each group

Not applicable. Due to the nature of our simulation-based study, participants incurred no harms.

19-i) Include privacy breaches, technical problems

We experienced no technical issues with the app during the whole study period.

19-ii) Include qualitative feedback from participants or observations from staff/researchers

"The questionnaire evaluating perceived stress and satisfaction scores was completed and returned by 100% of participants. Participants in groups A and B rated the overall perceived stress before the scenario to be 5.3 (95% CI 4.0 to 6.6) and 5.1 (95% CI 3.9 to 6.3), respectively (P = .78). During the scenario, the stress was rated to remain contained by the app users (4.8 [95% CI 3.4 to 6.2], P = .55), whereas it increased significantly for residents relying on the PALS pocket cards (mean 6.8 [95% CI 5.9 to 7.8], P = .014) compared to app users (P=.012). Satisfaction tended to be greater using the app (7.5 [95% CI 6.5 to 8.5] vs 5.9 [95% CI 4.4 to 7.4], respectively, P = .07)."

DISCUSSION

20) CONSORT: Trial limitations, addressing sources of potential bias, imprecision, multiplicity of analyses

20-i) Typical limitations in ehealth trials

"Our study has some limitations. First, it was conducted during a resuscitation simulation-based scenario rather than tested in real-life situations. However, high-fidelity simulation is an essential method to teach resuscitation skills and technologies that cannot be practiced during real CPR as the diversity among patients and their diseases makes such studies difficult to standardize in critical situations. The low occurrence of p-IHCA also limits the implementation of randomized trials in real life [33]. Moreover, standardizing the scenario and the environment helped to avoid effect modifiers by limiting the influence of undesired variables on the outcomes. Realism was achieved as reflected by the stress level experienced by participants, who considered the simulation as highly stressful as real CPR situations. Second, the 5-min app training was dispensed just before the scenario. In real life, the interval between training and actual use would probably be months. However, training with the app months before the study would have unblinded participants to its purpose and could have created a preparation bias. Third, the sample size limited stratified analyses to estimate the impact of PALS certification on the outcomes, but a recent study observed that improved adherence to AHA recommendations was not directly associated with PALS-trained providers [7].

Finally, we acknowledge that our findings might not be generalizable to providers with extensive CPR experience, such as pediatric emergency physicians. As only residents were assessed in this trial, further studies would be valuable to assess this assumption."

21) CONSORT: Generalisability (external validity, applicability) of the trial findings

21-i) Generalisability to other populations

"Inter-individual variance was also reduced with the app, suggesting a worthwhile benefit of its use by residents with various experience levels."

"This well correlates with the results of Hunt et al. who observed that despite the availability of AHA recommendations, 66% of pediatric residents failed to start compressions within 60 sec from the onset of a simulated pVT, 33% never started compressions, only 54% successfully defibrillated within 180 sec, and 7% never discharged the defibrillator [29]."

"This could potentially negatively affect patient outcome as choosing the wrong electrical therapy, drugs or algorithm in real life might impede the correct management of critically ill children and jeopardize their chance of survival."

"It would be interesting in further studies to determine whether this would translate into fewer deviations in shock doses in real life."

"In the present study, displaying the entire algorithm on a larger screen size of a tablet and paralleling stepwise patient-centered care guidance appeared to improve adherence to AHA guidelines and speed up skills, thus allowing residents to better manage simulated CPR. It would be interesting in further studies to assess this assumption with certified emergency physicians or paramedics in simulated and real-life in- or out-of-hospital environments."

"Finally, we acknowledge that our findings might not be generalizable to providers with extensive CPR experience, such as pediatric emergency physicians. As only residents were assessed in this trial, further studies would be valuable to assess this assumption."

21-ii) Discuss if there were elements in the RCT that would be different in a routine application setting

"it was conducted during a resuscitation simulation-based scenario rather than tested in real-life situations. However, high-fidelity simulation is an essential method to teach resuscitation skills and technologies that cannot be practiced during real CPR as the diversity among patients and their diseases makes such studies difficult to standardize in critical situations. The low occurrence of p-IHCA also limits the implementation of randomized trials in real life [33]."

"Realism was achieved as reflected by the stress level experienced by participants, who considered the simulation as highly stressful as real CPR situations."

"Second, the 5-min app training was dispensed just before the scenario. In real life, the interval between training and actual use would probably be months. However, training with the app months before the study would have unblinded participants to its purpose and could have created a preparation bias. Third, the sample size limited stratified analyses to estimate the impact of PALS certification on the outcomes, but a recent study observed that improved adherence to AHA recommendations was not directly associated with PALS-trained providers [7]. Finally, we acknowledge that our findings might not be generalizable to providers with extensive CPR experience, such as pediatric emergency physicians. As only residents were assessed in this trial, further studies would be valuable to assess this assumption."

22) CONSORT: Interpretation consistent with results, balancing benefits and harms, and considering other relevant evidence

22-i) Restate study questions and summarise the answers suggested by the data, starting with primary outcomes and process outcomes (use)

We investigated whether a mobile app developed as a guide to support and drive CPR providers in real-time through interactive pediatric advanced life support (PALS) algorithms would increase adherence to AHA guidelines and reduce the time to initiation of critical life-saving maneuvers compared to the use of PALS pocket reference cards. We found that a PALS-based mobile app designed for tablets to interactively support residents during pediatric CPR contributed to a shorter time to first and subsequent defibrillation attempts, lesser medication and defibrillation dose errors, as well as a better adherence to AHA recommendations, compared with the conventional PALS pocket reference cards. Taken together, our results suggest that residents are not following accurately AHA recommendations during pediatric CPR when only supported by PALS pocket cards.

22-ii) Highlight unanswered new questions, suggest future research

"A next step would be to determine in real-life studies whether this mobile app might benefit patients by improving the adherence and performance of residents to meet AHA resuscitation requirements in clinical practice."

Other information

23) CONSORT: Registration number and name of trial registry

"The trial received a declaration of no objection by Swissethics and the Geneva cantonal ethics committee as the purpose of the study was to examine the effect of the intervention on healthcare providers. For the same reason and according to the International Committee of Medical Journal Editors, a trial registration number was not required."

24) CONSORT: Where the full trial protocol can be accessed, if available

The full trial protocol can be obtained upon request to the Geneva University Hospitals.

25) CONSORT: Sources of funding and other support (such as supply of drugs), role of funders

Geneva University Hospitals are the owner of the app “Guiding Pad,” which is not available at the time of submission on the Google Play Store and Apple App Store. All authors declare no conflicts of interest. The present trial had financial support from the private foundation of Geneva University Hospitals (fund no. QS2-25). The funder of the study had no role in study design, data collection, data analysis, data interpretation, or writing of the report.

X26-i) Comment on ethics committee approval

"The trial received a declaration of no objection by Swissethics and the Geneva cantonal ethics committee as the purpose of the study was to examine the effect of the intervention on healthcare providers."

x26-ii) Outline informed consent procedures

"Written informed consent was obtained from each participant after full information disclosure prior to study participation."

X26-iii) Safety and security procedures

Data collected during the interventions were entered in Microsoft Excel spreadsheets by the investigators of the study, in anonymised form. The database was safely stored on secured hard disk drives and kept in locked cabinets, centralised at the Geneva Children's Hospital, Geneva, Switzerland. A password system was used to control access to the hard disk drives. Data collected on individuals were made anonymous. Only anonymised and summarized data were submitted as part of the statistical analysis. No directly or indirectly personal participant’s study informations were released outside of the study without a written permission of the participant. Any individual performance undergone during the resuscitation scenario remained confidential and was not communicated at the institutional level.

X27-i) State the relation of the study team towards the system being evaluated

Geneva University Hospitals are the owner of the app “Guiding Pad,” which is not available at the time of submission on the Google Play Store and Apple App Store. All authors declare no conflicts of interest.
