# Supplementary material for: The Impact of a Tablet App on Adherence to American Heart Association Guidelines During Simulated Pediatric Cardiopulmonary Resuscitation: Randomized Controlled Trial
Source: J Med Internet Res. 2020 May 27;22(5):e17792. doi: 10.2196/17792 (PMC7287744; doi:10.2196/17792)
Supplement: Multimedia Appendix 2 [file jmir_v22i5e17792_app2.docx]

**Multimedia Appendix 2**

**pVT resuscitation scenario**

The untimed portion of the simulation started by inviting the resident waiting outside the shock room to enter. When entering the room, a clinical statement to recognize the life-threatening condition of the patient, including his weight and age, was given by the study investigator as follows: “Here is a 25 kg, 7-year-old boy transferred by ambulance from a community-based hospital to our emergency department for managing an already confirmed severe myocarditis. The patient lost consciousness a few seconds ago and was immediately brought into the shock room. He is pale and not breathing. You are the team leader, please start CPR!”. At this moment, the timed scenario began. The participant had to recognize by themselves the previously settled cardiac rhythm (pVT) with the app (group A) or the PALS conventional pocket reference cards (group B). Monitoring alarms were activated to increase the realism. The scenario ran invariably until the manikin was defibrillated at the fourth shock and showed a subsequent return of spontaneous circulation.
