# Supplementary material for: The Impact of a Tablet App on Adherence to American Heart Association Guidelines During Simulated Pediatric Cardiopulmonary Resuscitation: Randomized Controlled Trial
Source: J Med Internet Res. 2020 May 27;22(5):e17792. doi: 10.2196/17792 (PMC7287744; doi:10.2196/17792)
Supplement: Multimedia Appendix 3 [file jmir_v22i5e17792_app3.docx]

# **Multimedia Appendix 3: 10-point Likert scales**

**Before the scenario starts:**

1. On a scale from 1 to 10, how stressed are you now:

Totally unstressed 1 2 3 4 5 6 7 8 9 10 Totally stressed

**At the end of the scenario:**

2. On a scale from 1 to 10, how stressed (maximum reached) were you during the scenario with the PALS reference cards:

Totally unstressed 1 2 3 4 5 6 7 8 9 10 Totally stressed

3. On a scale from 1 to 10, how stressed (maximum reached) were you during the scenario with the “Guiding Pad” app:

Totally unstressed 1 2 3 4 5 6 7 8 9 10 Totally stressed

4. On a scale from 1 to 10, how satisfied were you with your “Guiding Pad” experience?

Very dissatisfied 1 2 3 4 5 6 7 8 9 10 Very satisfied
