# Supplementary material for: The Impact of a Tablet App on Adherence to American Heart Association Guidelines During Simulated Pediatric Cardiopulmonary Resuscitation: Randomized Controlled Trial
Source: J Med Internet Res. 2020 May 27;22(5):e17792. doi: 10.2196/17792 (PMC7287744; doi:10.2196/17792)
Supplement: Multimedia Appendix 4 [file jmir_v22i5e17792_app4.docx]

**Table S1. Interrater agreement on outcome analyses.**

| **Outcome** | **Reviewer 1,** n/N (%) | **Reviewer 2**, n/N (%) | **Kappa coefficient** (95% CI) |
| --- | --- | --- | --- |
| 1^st^ defibrillation attempt |  |  |  |
| Wrong pVT rhythm identification | 10/26 (30.8) | 10/26 (30.8) | 1 (NA) |
| Wrong electrical therapy | 3/26 (11.5) | 3/26 (11.5) | 1 (NA) |
| Wrong shock energy dose | 3/26 (7.7) | 3/26 (7.7) | 1 (NA) |
| Do not resume chest compression immediately after shock | 1/26 (3.8) | 1/26 (3.8) | 1 (NA) |
| 2^nd^ defibrillation attempt |  |  |  |
| Wrong pVT rhythm identification | 9/26 (30.8) | 9/26 (30.8) | 1 (NA) |
| Wrong electrical therapy | 1/26 (3.8) | 1/26 (3.8) | 1 (NA) |
| Wrong shock energy dose | 3/26 (11.5) | 3/26 (11.5) | 1 (NA) |
| Do not resume chest compression immediately after shock | 1/26 (3.8) | 1/26 (3.8) | 1 (NA) |
| Epinephrine |  |  |  |
| Wrong drug dose | 1/26 (3.8) | 1/26 (3.8) | 1 (NA) |
| Wrong timing | 3/26 (11.5) | 3/26 (11.5) | 1 (NA) |
| 3^rd^ defibrillation attempt |  |  |  |
| Wrong pVT rhythm identification | 8/26 (26.9) | 8/26 (26.9) | 1 (NA) |
| Wrong electrical therapy | 1/26 (3.8) | 1/26 (3.8) | 1 (NA) |
| Wrong shock energy dose | 3/26 (11.5) | 3/26 (11.5) | 1 (NA) |
| Do not resume chest compression immediately after shock | 1/26 (3.8) | 1/26 (3.8) | 1 (NA) |
| Amiodarone (or lidocaine) |  |  |  |
| Wrong drug dose | 1/26 (3.8) | 1/26 (3.8) | 1 (NA) |
| Wrong timing | 4/26 (15.4) | 4/26 (15.4) | 1 (NA) |
| 4^th^ defibrillation attempt |  |  |  |
| Wrong pVT rhythm identification | 7/26 (26.9) | 7/26 (26.9) | 0.90 (0.70 to 1) |
| Wrong electrical therapy | 0/26 (0) | 0/26 (0) | 1 (NA) |
| Wrong shock energy dose | 0/26 (0) | 0/26 (0) | 1 (NA) |
| Do not resume chest compression immediately after shock | 0/26 (0) | 0/26 (0) | 1 (NA) |
| Wrong PALS sequence | 11/26 (42.3) | 11/26 (42.3) | 1 (NA) |
| n: number of errors; N: number of opportunities to commit errors; 95% CI: 95% confidence interval | | | |

The table details the interrater agreement on PALS algorithm for pVT reviews by error type following the optimal sequence of PALS interventions, expressed as Cohen kappa coefficients. Poor reliability was defined as a kappa coefficient of <.40, weak reliability as .40-.59, moderate reliability as .60-.79, strong reliability as .80-.90, and almost perfect as >.90.
