# Supplementary material for: The Impact of a Tablet App on Adherence to American Heart Association Guidelines During Simulated Pediatric Cardiopulmonary Resuscitation: Randomized Controlled Trial
Source: J Med Internet Res. 2020 May 27;22(5):e17792. doi: 10.2196/17792 (PMC7287744; doi:10.2196/17792)
Supplement: Multimedia Appendix 5 [file jmir_v22i5e17792_app5.docx]

**Table S2. Bland & Altman and intra-class correlation coefficient on outcome analyses.**

| **Outcome**  [seconds] | **Reviewer 1**  mean (SD) | **Reviewer 2**  mean (SD) | **Mean difference**  (95% CI) P-value | **Limits of agreement** | **ICC** |
| --- | --- | --- | --- | --- | --- |
| Start chest compression | 40.31 (52.25) | 40.35 (52.22) | .038 (-.39 to .31), P=.82 | -1.75 to 1.67 | 1.00 (1.00 to 1.00) |
| 1st defibrillation attempt | 166.5 (74.95) | 166.3 (75.07) | .12 (-.093 to .32), P=.12 | -0.90 to 1.13 | 1.00 (1.00 to 1.00) |
| 2nd defibrillation attempt | 300.6 (79.82) | 300.3 (79.85) | .23 (-.006 to .47), P=.056 | -0.92 to 1.38 | 1.00 (1.00 to 1.00) |
| IO route | 185.1 (58.62) | 184.9 (58.63) | .19 (-.29 to .68), P=.42 | -2.16 to 2.55 | 1.00 (1.00 to 1.00) |
| Epinephrine | 278.1 (77.90) | 278.3 (77.96) | .19 (-.59 to .20), P=.33 | -2.11 to 1.73 | 1.00 (1.00 to 1.00) |
| 3rd defibrillation attempt | 488.8 (129.2) | 488.8 (129.1) | .00 (-.23 to .23), P=1.00 | -1.11 to 1.11 | 1.00 (1.00 to 1.00) |
| Amiodarone | 526.9 (149.2) | 526.8 (149.4) | .077 (-.28 to .44), P=.66 | -1.67 to 1.82 | 1.00 (1.00 to 1.00) |
| 4th defibrillation attempt | 643.4 (160.4) | 643.6 (160.9) | .19 (-.59 to .20), P=.33 | -2.11 to 1.73 | 1.00 (1.00 to 1.00) |
| SD: standard error; ICC: intra-class correlation coefficient; 95% CI: 95% confidence interval. | | | | | |

The table details the values for the Bland and Altman analyzes as well as the intra-class correlation coefficients for the interrater reliability between observers 1 and 2 at the time of shocks or drug doses delivered by the 26 residents (see Figure S1).

CPR: cardiopulmonary resuscitation; IO: intraosseous; SD: standard deviation; ICC: intra-class correlation coefficient; 95% CI: 95% confidence interval.
