# Supplementary material for: The Impact of a Tablet App on Adherence to American Heart Association Guidelines During Simulated Pediatric Cardiopulmonary Resuscitation: Randomized Controlled Trial
Source: J Med Internet Res. 2020 May 27;22(5):e17792. doi: 10.2196/17792 (PMC7287744; doi:10.2196/17792)
Supplement: Multimedia Appendix 6 [file jmir_v22i5e17792_app6.docx]

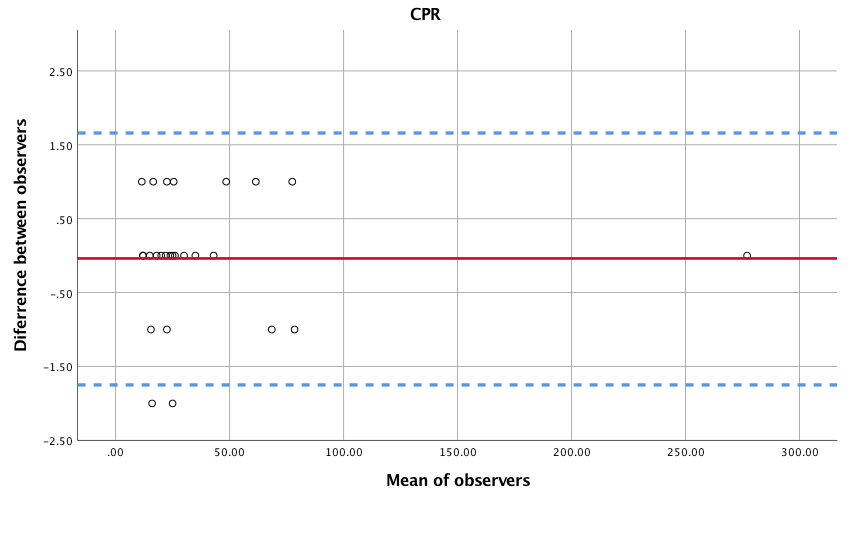

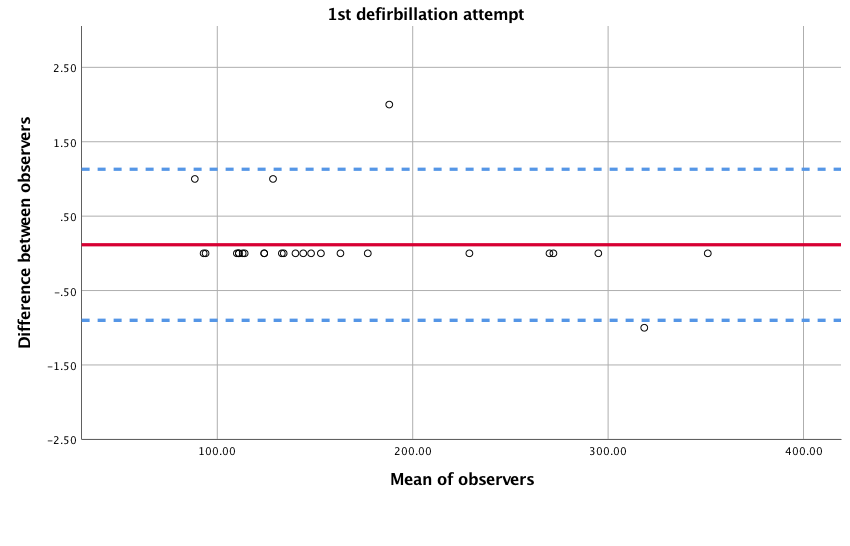


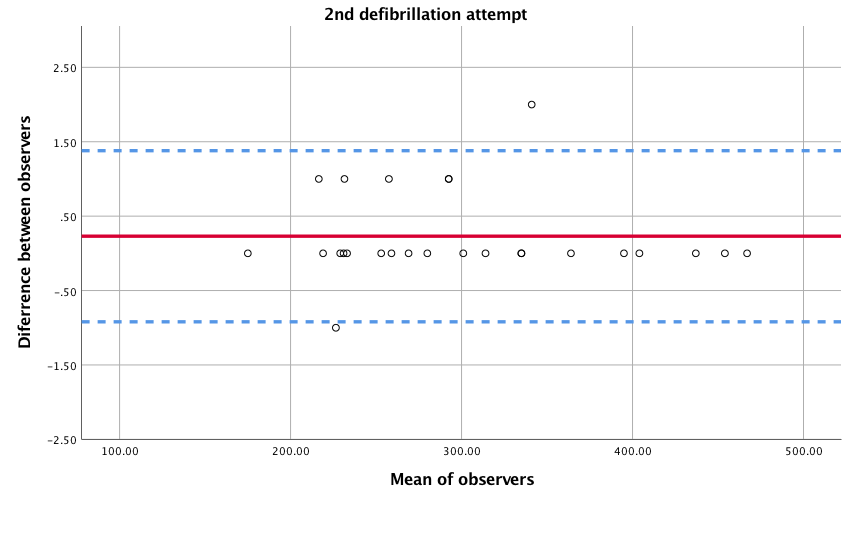

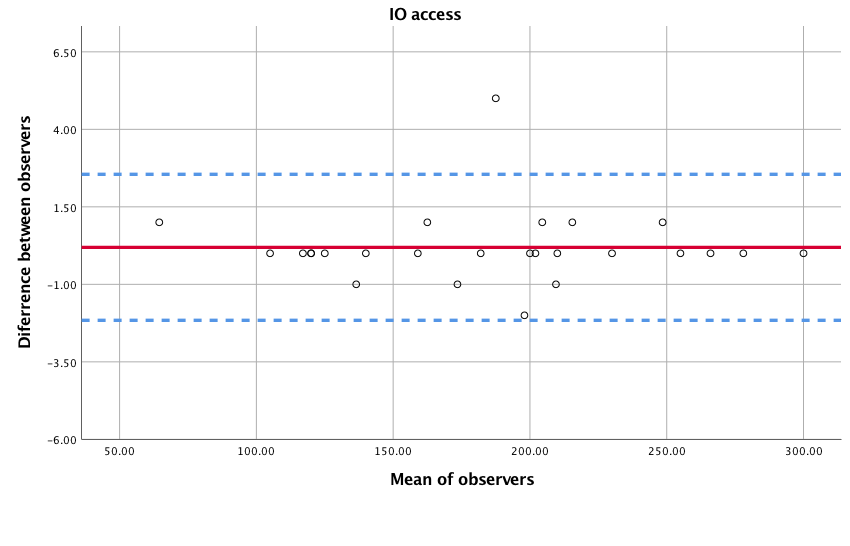


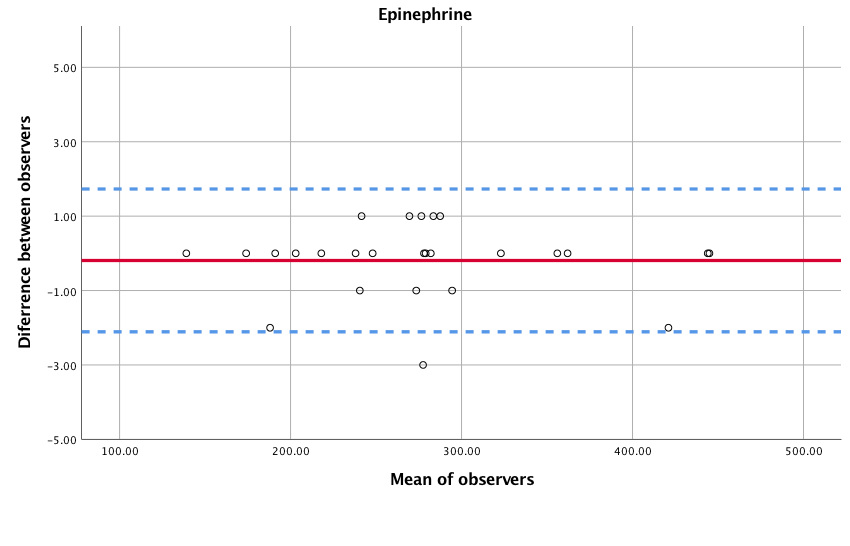

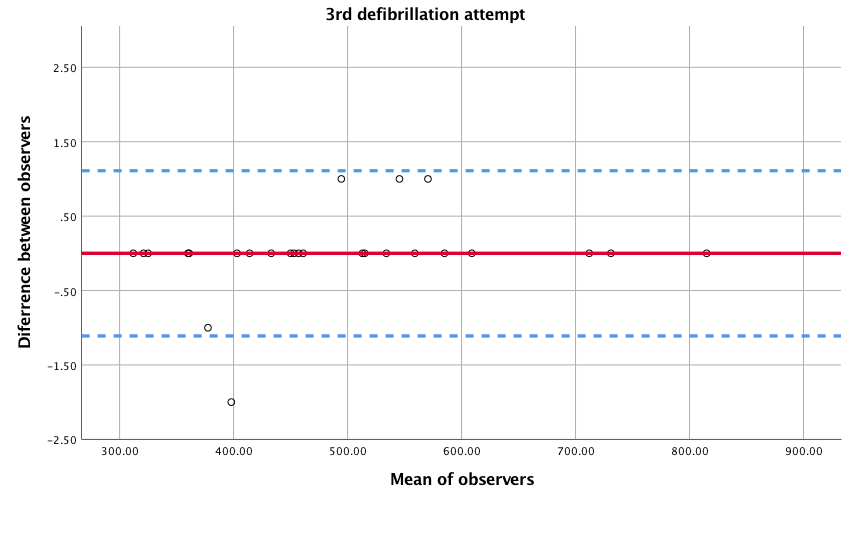


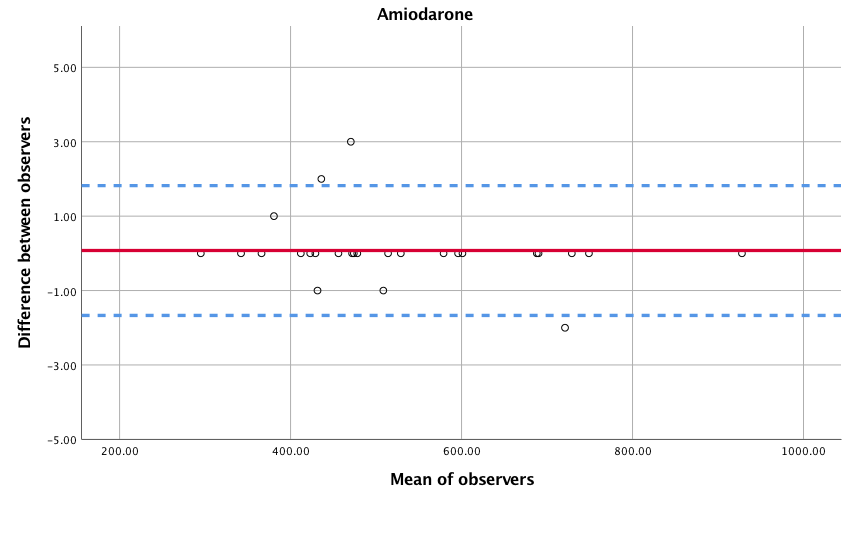

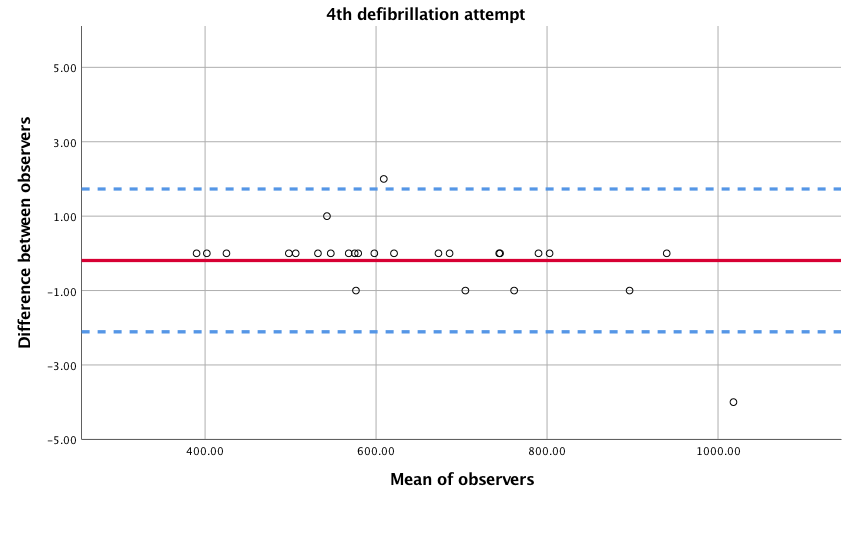


**Figure S1. Bland and Altman analysis of pVT algorithm review.** The Bland-Altman plots illustrate the difference between observers 1 and 2 in the time to shocks or drug doses delivery by 26 residents, plotted against the mean value of both observers (see values in Table S2). Solid red lines denote the mean difference. Dashed blue lines denote upper and lower limits of agreement from -1.96 SD to +1.96 SD of the mean. CPR: cardiopulmonary resuscitation; IO: intra-osseous; SD: standard error; ICC: intra-class correlation coefficient; 95% CI: 95% confidence interval.
