# Supplementary material for: The Impact of a Tablet App on Adherence to American Heart Association Guidelines During Simulated Pediatric Cardiopulmonary Resuscitation: Randomized Controlled Trial
Source: J Med Internet Res. 2020 May 27;22(5):e17792. doi: 10.2196/17792 (PMC7287744; doi:10.2196/17792)
Supplement: Multimedia Appendix 8 [file jmir_v22i5e17792_app8.docx]

**
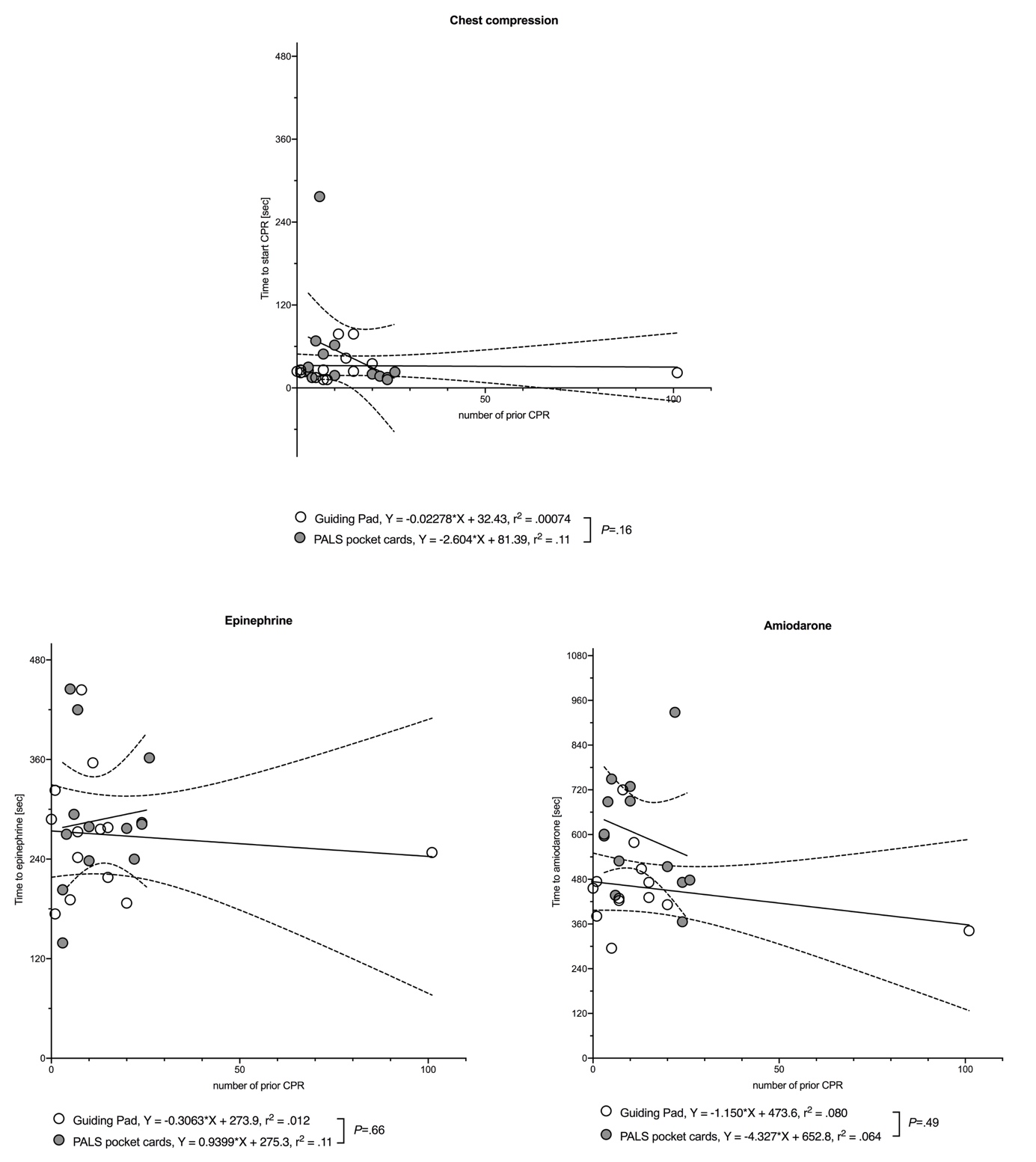
**

**Figure S3. Association between time to chest compression or drug delivery and number of prior CPR.** Data are regression line (solid) with 95% CI (dashed lines). P values and r^2^ values are based on simple linear regression analysis. White (Guiding Pad) and grey (PALS pocket cards) open circles denote each individual value.
